# Treg engage lymphotoxin beta receptor for afferent lymphatic transendothelial migration

**DOI:** 10.1038/ncomms12021

**Published:** 2016-06-21

**Authors:** C. Colin Brinkman, Daiki Iwami, Molly K. Hritzo, Yanbao Xiong, Sarwat Ahmad, Thomas Simon, Keli L. Hippen, Bruce R. Blazar, Jonathan S. Bromberg

**Affiliations:** 1 Center for Vascular and Inflammatory Diseases, University of Maryland School of Medicine, Baltimore, Maryland 21201, USA; 2 Department of Microbiology and Immunology, University of Maryland School of Medicine, Baltimore, Maryland 21201, USA; 3 Department of Surgery, University of Maryland School of Medicine, Baltimore, Maryland 21201, USA; 4 Division of Blood and Marrow Transplantation, Department of Pediatrics, University of Minnesota Cancer Center, Minneapolis, Minnesota 55455, USA

## Abstract

Regulatory T cells (Tregs) are essential to suppress unwanted immunity or inflammation. After islet allo-transplant Tregs must migrate from blood to allograft, then via afferent lymphatics to draining LN to protect allografts. Here we show that Tregs but not non-Treg T cells use lymphotoxin (LT) during migration from allograft to draining LN, and that LT deficiency or blockade prevents normal migration and allograft protection. Treg LTαβ rapidly modulates cytoskeletal and membrane structure of lymphatic endothelial cells; dependent on VCAM-1 and non-canonical NFκB signalling via LTβR. These results demonstrate a form of T-cell migration used only by Treg in tissues that serves an important role in their suppressive function and is a unique therapeutic focus for modulating suppression.

## Abstract (web summary)

Lymphotoxin regulates lymphoid organ architecture and adhesion molecules involved in lymphocyte trafficking. Here the authors show that lymphotoxin produced by regulatory T cells promotes their migration to the draining lymph nodes by engaging its cognate receptor on lymphatic endothelial cells.

## Graphical abstract

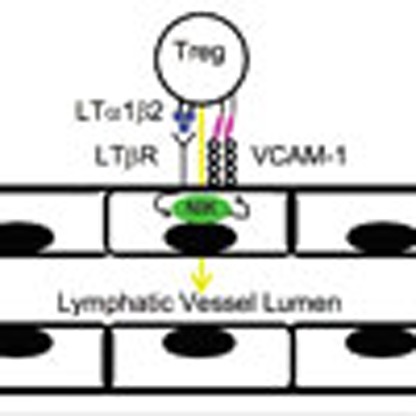

Regulatory T cells (Tregs) help maintain immunological tolerance and resolve inflammation following infections^1^. Treg induction or transfer is of interest for treatment of a variety of diseases. Treg must migrate to both grafts and lymph nodes (LN) to promote allograft acceptance^2^,^3^,^4^. We previously reported that Tregs migrate from blood to islet allografts, then to afferent lymphatics and the draining LN^2^, and that Treg migration from graft to LN was required for optimal graft survival. Others found that Tregs are the major lymphocyte subset migrating from inflamed skin during contact hypersensitivity and that such migration is involved in regulating inflammation^5^. Thus, Treg migration to draining LN via lymphatics is a normal part of the inflammatory response and important in inflammatory resolution.

In contrast to migration from blood to LN or non-lymphoid tissues, lymphocyte migration from tissues to LN via afferent lymphatics is incompletely understood. The most extensive literature on lymphatic migration regards dendritic cells (DCs)^6^,^7^,^8^, with less known about the migration of T cells^9^, or other cells, such as neutrophils^10^. In mice, DCs follow CCL21 gradients to lymphatics using the chemokine receptor CCR7, where they enter lymphatic capillaries via flaps between overlapping lymphatic endothelial cells (LECs) in a process that does not require integrins or proteolysis^11^,^12^.

It had been thought that, like DCs, T cells use CCR7 to exit tissue and access lymphatics^13^,^14^. However, recent work found that T cells and DCs use CCR7 differently during migration from afferent lymph to LN, and T cells do not need CCR7 to enter LN from lymph^15^. Others report that CD4+ T cells do not require CCR7 to exit tissue, enter lymph and infiltrate LN while CD8+ T cells do^16^. These conflicting reports underscore how little is known about the mechanisms governing T-cell afferent lymph migration. It is also not known if Tregs rely on the same or different mechanisms as non-Treg or DC for lymphatic migration or tissue egress.

Lymphotoxins (LTs) are cytokines related to tumour necrosis factor alpha (TNFα), and function in organizing and maintaining lymphoid organs, and as cytotoxic effector molecules^17^. There are two LT subunits, soluble α and membrane-bound β, primarily found as a soluble homotrimer of α (LTα3) that binds TNF receptors, or a membrane-bound heterotrimer (LTα1β2) that interacts with the LT β receptor (LTβR)^18^. LTα1β2 is expressed on activated T, B and natural killer cells^18^,^19^, and interacts with LTβR on DC, monocyte lineage cells and stromal cells^17^. Murine array data suggest that Tregs express elevated levels of LTα compared with other T cells^20^. LTβR is required for proper migration of autoreactive T cells during thymic negative selection^21^, and B cell LTα1β2 contributes to a positive feedback loop that induces CXCL13 in follicular DCs^22^. LT, likely expressed by DCs, promotes the homeostatic maintenance of high endothelial venules (HEV) adhesion molecule and chemokine expression^23^,^24^, yet LTαβ expressed by T cells has not been described to be directly involved in their migration.

Here we report that Tregs use LTαβ to stimulate LTβR on lymphatic endothelium for migration to LN via afferent lymphatics. This interaction is not used by non-Treg T cells and is not required for Treg migration from blood through HEV into the LN, or from LN into efferent lymphatics. Tregs, but not non-Treg CD4+ T cells, induce rapid growth of lamellipodia-like projections from LEC but not blood endothelial cells (BECs) in an LTαβ–LTβR-dependent manner. These cytoplasmic membrane projections correlate with altered movements and transmigration patterns of Treg as they travel across LEC. These results demonstrate a novel form of T-cell migration utilized by Treg in tissues that serves an important role in their suppressive function and is a unique target for modulating suppression.

## Results

### LT regulates Treg suppressor function *in vivo*

**Figure 1:**
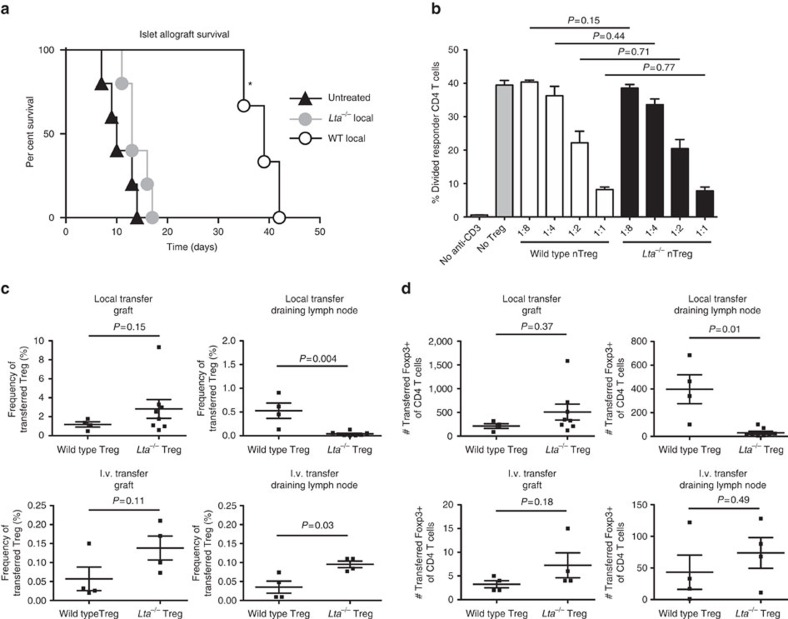
LT regulates Treg suppressor function *in vivo* but not *in vitro.* (**a**) Survival curves for BALB/c islets transplanted into diabetic C57BL/6J recipients monitored by blood glucose. Five untreated from two pooled experiments, five *Lta*^−/−^ Treg recipients from two pooled experiments, six WT Treg recipients from three pooled experiments. For multiple long-rank comparisons among three curves *P*=0.0167 was considered significant for individual comparisons. *Indicates WT is different than both *Lta*^−/−^ and untreated by log-rank comparison (*P*=0.0007) and mean survival time: 38.7±1.3 days (s.e.m.), *n*=6 WT versus 14.00±1.1 days *n*=5 *Lta*^−/−^, *P*<0.01, and 38.7±1.3 days WT versus 10.6±1.3 days *n*=5 untreated, *P*<0.01, Tukey post tests of one-way analysis of variance. (**b**) *In vitro* suppression showing per cent of conventional CD4 responder T cells that divided under various conditions. Triplicate wells in each of two experiments, pooled. Statistics from unpaired Student's *t*-tests. (**c**,**d**) Islet and draining LN migration. Scatter plots show % (**c**) or number (**d**) of all CD4 T cells in indicated organ that are transferred Treg. Four recipients from two experiments for WT and eight from three experiments for *Lta*^−/−^ Treg transfer. Statistics from Mann–Whitney non-parametric tests (**b**–**d**). Error bars are s.e.m.

To determine if LT was important for Treg function, natural Tregs (nTregs) were isolated from young, naive wild-type (WT) C56BL/6J mice or mice deficient in LT α (*Lta*^−/−^), and transferred into diabetic C56BL/6J recipients under the kidney capsule with BALB/c pancreatic islets. As previously shown^2^, local transfer of WT nTreg promoted substantial survival prolongation of islet allografts relative to no nTreg transfer by mean survival time (MST) and by log-rank survival curve comparison (Fig. 1a). In contrast, local transfer of *Lta*^−/−^ nTreg resulted in no increase in MST or difference by log-rank comparison relative to no transfer and a decrease in survival compared with WT nTreg by MST and log-rank survival comparison (Fig. 1a).

We previously demonstrated that Treg must migrate sequentially from islet allografts to draining LN to prolong islet survival^2^. Protection was prevented by blocking Treg entry into the graft or egress from the graft into draining LN. As the Tregs were placed under the kidney capsule with the islets in these experiments (Fig. 1a), this left several possible reasons for the defective protection afforded by *Lta*^−/−^ nTreg: *Lta*^−/−^ nTreg could be defective in suppression; have a proliferative or survival defect; and/or fail to traffic to draining LN, a step essential for islet allograft protection^2^. To distinguish these possibilities, Treg suppression of CD4+ T-cell proliferation was assessed *in vitro*. WT and *Lta*^−/−^ nTreg equally suppressed proliferation of conventional CD4+ T cells to anti-CD3ɛ monoclonal antibody (mAb), throughout a range of suppressor to responder ratios (Fig. 1b). There were also no significant differences in expression of *Il10* (interleukin 10), *Tgfb1*, *Prf1* (perforin), *Gzmb* (granzyme B), *Ifng* (interferon gamma), *Cd39*, *Cd73* and *Ctla4* by real-time quantitative reverse transcription–PCR (qRT–PCR) between WT and *Lta*^−/−^ nTreg (Supplementary Fig. 1a). *Lta*^−/−^ nTreg had no defect in proliferation or viability compared with WT nTreg when stimulated with anti-CD3ɛ and anti-CD28 *in vitro* (Supplementary Fig. 1b).

Together, these data suggested *Lta*^−/−^ nTreg had no defect in suppressor function or predisposition to apoptosis. Treg frequency (Fig. 1c) and number (Fig. 1d) in grafts 4 days after local co-transfer was assessed. There was a trend for higher frequencies and numbers of *Lta*^−/−^ nTreg of total CD4+ T cells in grafts compared with WT nTreg (Fig. 1c,d). This suggested that *LTα*^−/−^ nTreg did not have a survival defect *in vivo*. Indeed, there was no difference in the ratio of anti-apoptotic *Bcl2* to pro-apoptotic *Bcl2l11* (*Bim)* between WT and *Lta*^−/−^ Treg (Supplementary Fig. 1a). Treg frequency in draining LN was also measured. *Lta*^−/−^ nTreg were significantly less frequent in the graft-draining renal LN compared with WT nTreg (Fig. 1c,d). When nTreg were transferred intravenously (i.v.) instead of locally, *Lta*^−/−^ nTregs were present at a similar percentage and number as WT nTreg in the graft (Fig. 1c,d), while *Lta*^−/−^ nTreg representation in draining LN was slightly higher (Fig. 1c,d). This suggests that *Lta*^−/−^ nTreg had no defect in entering the graft or draining LN from blood relative to WT. These results indicated that *Lta*^−/−^ nTreg had normal suppressive function *in vitro*, no gross survival defect, but a profound defect in migration from tissue to draining LN.

### Tregs express more LTαβ compared with other T-cell subsets

**Figure 2:**
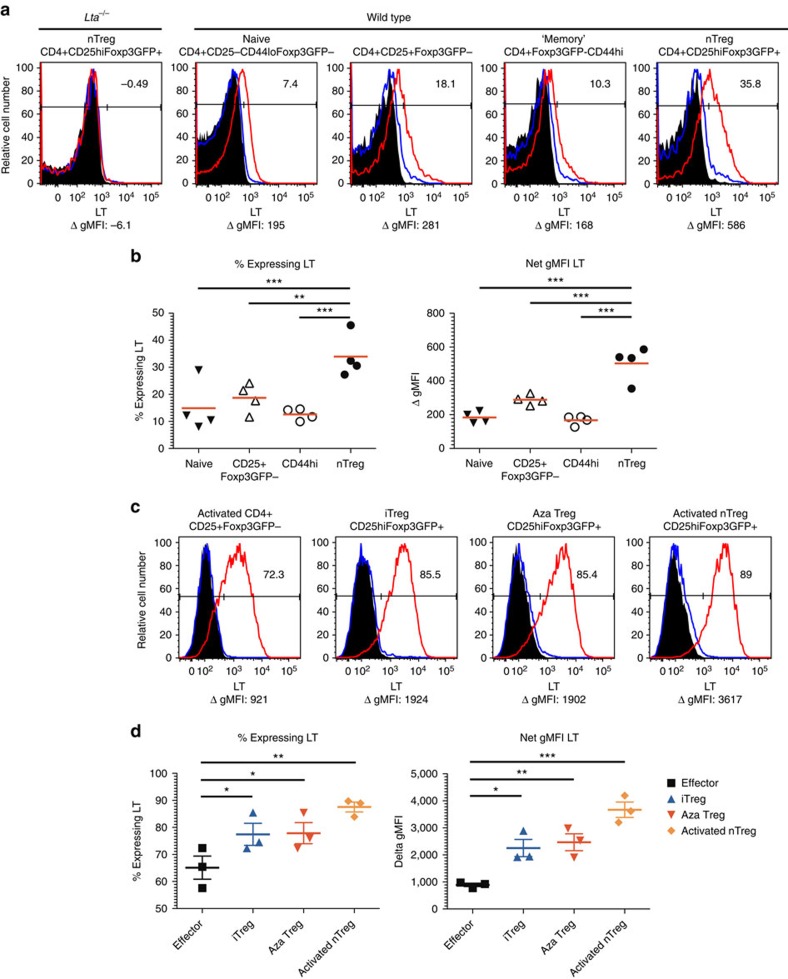
Tregs express more LTαβ than non-Treg. (**a**,**c**) Flow cytometry for LT on freshly isolated (**a**) or 5-day cultured (**c**) CD4+ T cells from LN and spleen of Foxp3GFP (**a**,**c**) or *Lta*^−/−^ Foxp3GFP mice (**a**) as indicated. Histograms gated on indicated populations. Black histograms: secondary antibody only; blue histograms: MOPC21 control primary; red histograms: LTβRIg stained. Numbers in plots indicate % specific positive staining in LTβRIg-stained minus MOPC21-stained samples. Numbers below are geometric mean fluorescence intensity (gMFI) of LTβRIg stained minus MOPC21 stained. Results typical of four experiments in **a** and three in **c**. (**b**,**d**) Summary data for %LT+ and gMFI LT from **a** and **c**, respectively. *P<0.05, **P<0.01, ***P<0.001 by repeated measures analysis of variance with Tukey post tests in **b** and **d**.

To determine if LT might be particularly relevant to Treg, the expression of various LT family members was assessed by flow cytometry and qRT–PCR. Using LTβRIg to detect surface LT, freshly isolated nTreg expressed more LT than naive CD4+CD25−CD44loFoxp3− T cells, recently activated CD4+CD25+Foxp3− T cells, or CD4+CD44hi memory T cells as judged by both geometric mean fluorescence intensity and per cent positive staining (Fig. 2a,b). *Lta*^−/−^ nTreg did not bind LTβRIg (Fig. 2a). For Treg culture, naive CD4 T cells and nTreg were flow sorted from Foxp3GFP reporter mice and stimulated with anti-CD3 and interleukin (IL)-2 (activated CD4+ and nTreg); anti-CD3, IL-2 and TGF-β1 (induced Treg or iTreg); or anti-CD3, IL-2, TGF-β1 and 5-Aza-2′-deoxycytidine (Aza Treg), which we previously showed generated an epigenetically demethylated iTreg with similarities to nTreg^25^. Cultured Treg subsets all expressed more LT than activated CD4+CD25+ non-Treg (Fig. 2c,d), consistent with qRT–PCR results, which also showed that none of the Treg expressed substantial *Ltbr* (LT β receptor), LT family members *Tnfrsf14* (herpes virus entry mediator, *Hvem*), or *Tnfsf14* (*LIGHT*) (Supplementary Fig. 1c). These data suggested that LTα1β2 or LTα3 might have differing importance to the function or trafficking of Treg and non-Treg.

It was possible that *Lta*^−/−^ Treg had altered expression of molecules required for T-cell trafficking, and that the effect on migration was not directly related to LT. However, expression of LFA-1 (CD11a/CD18), CD31, α4β1 (VLA-4), α4β7, αEβ7 and αV integrins by flow and *Ccr7* and *S1pr1* by qRT–PCR revealed no differences between WT and *Lta*^*−/−*^ (Supplementary Fig. 2). This implicated LTα itself as likely involved in and important for Treg migration to LN via lymphatics.

### Treg use LTαβ–LTβR for *in vivo* migration into lymphatics

**Figure 3:**
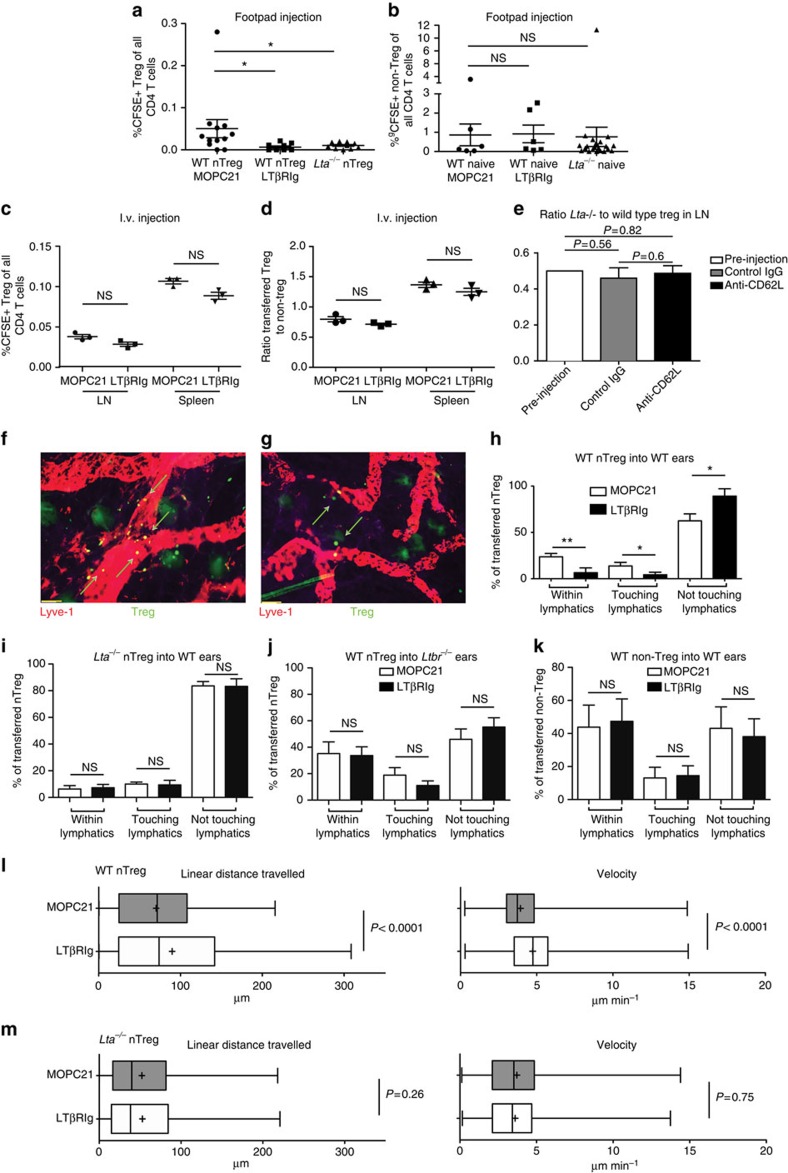
Tregs use LTαβ–LTβR for *in vivo* migration into lymphatics. Resting WT C57BL/6J or *Lta*^−/−^ CD25+CD4+ cells magnetic bead-enriched, CFSE-labelled, treated with LTβRIg or MOPC21, washed and injected into footpads (**a**,**b**), blood (**c**,**d**) or ear pinnae (**f**–**k**). Popliteal LN and spleens procured 12 h later and analysed by flow cytometry. In all, 9–12 mice per cell type from 3–4 experiments in **a**; 6–22 mice from 2–4 experiments in **b**; 3 mice per group from 1 experiment representative of 2 (**c**,**d**). (**e**) Ratio e670 dye-labelled *Lta*^−/−^ nTreg to CFSE-labelled WT nTreg in pre-injection mixture, or after i.v. injection followed by anti-CD62L or control mAb 18 h later. LN removed and analysed by flow cytometry 18 h later. *P* values from Wilcoxan signed rank test for pre-injection to control IgG and anti-CD62L; Student's *t*-test for control IgG versus anti-CD62L. In all, 1 pre-injection sample, 6 LN from control IgG treated animals and 9 LN from anti-CD62L-treated animals from 1 experiment, representative of 2. (**f**,**g**) Photomicrograph from ear pinna whole mount. LYVE-1+, lymphatics red; CFSE+ nTreg, green; × 20. Scale bar, 50 μm. MOPC21-treated (**f**); LTβRIg-treated (**g**). Green arrows indicate Treg in lymphatics (**f**) not in lymphatics (**g**). (**h**–**k**) Summary of ear pinnae data indicating relationship of transferred T cells to lymphatics. In all, 20–250 cells per field from 18–19 × 20 objective fields per condition derived from 9 ears per condition from 3 independent experiments in **h**; and 15–200 cells per × 20 objective field from 4–10 fields from 3 ears per condition in 1 experiment each representative of 2 experiments in **i**–**k**. (**l**,**m**) Summary of Treg movement in ears during 30 min of image acquisition. In all, 1,611 MOPC21- and 3,490 LTβRIg-treated WT nTreg, and 641 MOPC21- and 1,345 LTβRIg-treated *Lta*^−/−^ nTreg tracked using Volocity 6.1.1. Results pooled from one × 20 field per ear, 2–3 ears per condition from 2–3 independent experiments. *P<0.05, **P<0.01 Kruskal–Wallis and Dunn's multiple comparison (**a**,**b**), Student's *t*-test (**c**,**d**,**h**–**m**). (**a**–**k**) Error bars are s.e.m. (**l**,**m**) Box and whiskers plots showing minimum, maximum, mean (+), median (bar), 25th and 75th percentiles. NS, not significant.

*Lta*^−/−^ Treg lack both LTα3 and LTα1β2. LTα3 interacts with TNF receptors I and II and LTα1β2 with LTβR. Given the known role of LTβR in development and homeostasis of lymphatic endothelium and lymphoid organs^17^ we hypothesized that disruption of LTα1β2/LTβR interactions was responsible for the migration defect. To test this, the LTβRIg fusion protein of LTβR with mouse IgG1 was used in the lymphatic migration model of footpad to draining popliteal LN^26^. LTβRIg blocks interactions of either LIGHT or LTα1β2 with LTβR. Carboxyfluorescein succinimidyl ester (CFSE)-labelled nTreg were pretreated with LTβRIg or control isotype-matched MOPC21 antibody, washed, injected into hind footpads and draining popliteal LN examined 12 h later. LTβRIg pretreatment or use of *Lta*^−/−^ nTreg resulted in large and significant decreases in nTreg migration to the draining LN (Fig. 3a). Migration of naive non-Treg CD4+ T cells to the popliteal LN was unaffected by LTβRIg or genetic ablation of *Lta* (Fig. 3b), and migration of CD8 T cells from the footpad to the draining LN was also unaffected by LTβRIg (Supplementary Fig. 3a), indicating that the effect was specific for Treg. It was also possible that *Lta*^−/−^ Treg migrated through lymphatics normally but did not enter LN normally from lymph. However, fluorescent microscopy of popliteal LN from mice that received footpad injections of WT or *Lta*^−/−^ Treg revealed that when detectable, *Lta*^−/−^ Treg were visible in the LN parenchyma and not the subcapsular sinus, and were distributed normally (Supplementary Fig. 3b–d). Because Treg do not express *Tnfsf14/LIGHT* or *Ltbr* (Supplementary Fig. 1c), together these results showed that blocking interactions between LTα1β2 on Treg and LTβR expressed by other cells prevents migration into lymphatics and draining LN. LTβRIg did not affect Treg or naive CD4+ T-cell migration from blood into lymphoid organs, as there were no significant changes in the migration of LTβRIg pretreated Treg from blood into LN or spleen after i.v. injection (Fig. 3c), and the ratio of Treg to co-transferred naive non-Treg CD4+ T cells was unaltered by LTβRIg (Fig. 3d). These data also argue against an Fc-mediated inhibition of Treg migration due to increased binding of LTβRIg to Treg compared with non-Treg.

To determine if LN egress was also regulated by LT, a mixture of WT and *Lta*^−/−^ nTreg and non-Treg CD4+ T cells was co-transferred iv into WT mice, 18 h later recipients were treated with anti-CD62L mAb to prevent further LN entry via HEV, and another 18 h later LN and spleen were analysed by flow cytometry. Importantly, the ratio of *Lta*^−/−^ to WT nTreg did not differ between pre-injection and control LN, providing further evidence that Treg entry into LN from blood was not regulated by Treg expression of LT (Fig. 3e). Furthermore, if Treg-expressed LT were required for Treg LN egress, anti-CD62L should result in a higher ratio of *Lta*^−/−^ to WT Treg than in control animals. However, there were no differences in the ratio of *Lta*^−/−^ to WT Treg between control and anti-CD62L-treated LN (Fig. 3e). Thus, LT on Treg did not regulate Treg LN egress.

The ear pinna model of lymphatic migration was utilized to visualize T-cell encounters with lymphatics. LTβRIg pretreatment of WT nTreg resulted in a lower percentage of Treg within or touching the lymphatics and a greater percentage not touching the lymphatics compared with control treatment (Fig. 3f–h). In contrast *Lta*^−/−^ nTreg (Fig. 3i), WT nTreg transferred into *Ltbr*^−/−^ ears (Fig. 3j) and non-Treg CD4+ T cells (Fig. 3k) were not affected by LTβRIg. We also tracked the *in situ* movement of live Treg in ear explants. Treg in the vicinity of lymphatics moved farther and faster when treated with LTβRIg (Fig. 2l and Supplementary Movies 1 and 2). In contrast, movement of *Lta*^−/−^ Treg in ear explants was not affected by treatment with LTβRIg (Fig. 2m and Supplementary Movies 3 and 4). These observations were consistent with an essential role for Treg LTα1β2 interactions with LTβR in normal interstitial and/or lymphatic migration, and collectively, these data indicated that nTreg and iTreg, but not non-Treg, CD4+ T-cell migration into lymphatic vessels depended on Treg LTα1β2 interactions with LTβR on stromal or lymphatic cells.

### Tregs use LTαβ–LTβR for transmigration across LEC *in vitro*

**Figure 4:**
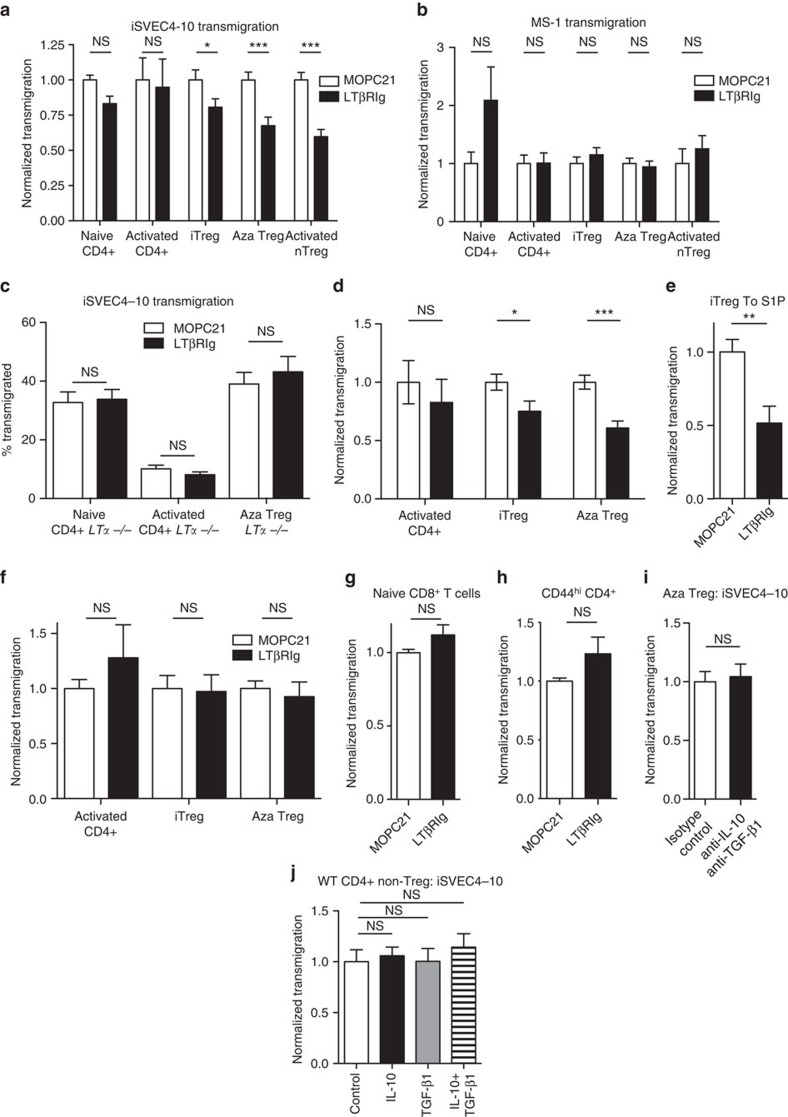
Treg uses LTαβ–LTβR for *in vitro* transmigration across a LEC line *in vitro*. (**a**–**j**) Indicated T cells migrated across iSVEC4-10 (**a**,**c**–**e**,**g**–**j**), MS-1 (**b**), plastic (**f**), for 4 h to CCL19 (**a**–**c**,**f**–**j**), CCL5 (**d**) or S1P (**e**) in the presence of LTβRIg or control MOPC21. Migrated T cells in lower wells enumerated and transmigration normalized to MOPC21 shown (**a**–**j**). A total of 9–15 individual transwells per condition from 3–4 experiments (**a**–**d**,**f**), 8 individual transwells per condition from 2 experiments (**e**), 10 individual transwells per condition pooled from 2 experiments (**g**), 5 individual transwells per condition from 2 experiments (**h**), 8 individual transwells per condition from 2 experiments (**i**) and 7 individual transwells per condition from 2 experiment (**j**). *P<0.05, **P<0.01, ***P<0.001 from unpaired two-tailed Student's *t*-tests (**a**–**i**) and Bonferroni multiple comparison tests in **j**. Error bars are s.e.m. NS, not significant.

We hypothesized that Treg LTα1β2 interaction with LEC LTβR was an important step in lymphatic migration. Since there were potentially several cell types in tissues *in vivo* that could express LTβR, we used a previously developed *in vitro* model to make definitive conclusions about the relevant cells and interactions. We assayed Treg transmigration across the SVEC4-10 LEC line and MS-1 BEC line^26^. These cells were grown on transwell chamber inserts to produce confluent monolayers, T cells were transmigrated across them and migrated cells in the lower chamber counted. Migration across lymphatic endothelium proceeds only from the basal to the luminal side of the endothelium, but not in the reverse direction^26^, so SVEC4-10 were grown on the lower sides of transwell inserts (Supplementary Fig. 4). In this orientation, SVEC4-10 layers are referred to as iSVEC4-10 for ‘inverted', and T-cell migration through these layers proceeded in the basal to luminal direction (Supplementary Fig. 4b). LTβRIg reduced transmigration of all Treg subsets across iSVEC4-10 to CCL19 (Fig. 4a). In contrast, migration of naive or activated non-Treg CD4+ T cells across iSVEC4-10 was not affected. LTβRIg did not block migration of any cell type across MS-1 (Fig. 4b). As expected, *Lta*^−/−^ Treg migration across iSVEC4-10 was unaltered by LTβRIg, indicating its specificity (Fig. 4c). These data were in concordance with *in vivo* footpad, ear pinna, islet allograft and blood to LN migration results (Figs 1c and 3a–l). Treg migration across iSVEC4-10 to CCL5 (Fig. 4d) and sphingosine-1-phosphate (Fig. 4e) was also inhibited by LTβRIg, indicating the effect was not unique to a single chemo-attractant or receptor. Treg migration across plastic was not blocked by LTβRIg, further proving specificity and suggesting the effects of LTβRIg were not Treg intrinsic, but depended on altering Treg interactions with SVEC4-10 (Fig. 4f). Migration of CD8 T cells, and memory CD44^hi^ CD4 T cells were unaffected by LTβRIg (Fig. 4g,h). Also, blockade of IL-10 and TGF-β1 with antibodies did not alter Treg migration across iSVEC4-10 to CCL19, nor did addition of IL-10 and/or TGF-β1 to CD4+ non-Treg alter their migration across iSVEC4-10 to CCL19 (Fig. 4i,j). Together these results indicated that Treg but not non-Treg migration across lymphatic but not blood endothelium depended in part on the interaction of Treg LTα1β2 with LEC-expressed LTβR.

### Treg migration across iSVEC4-10 is regulated by NIK

**Figure 5:**
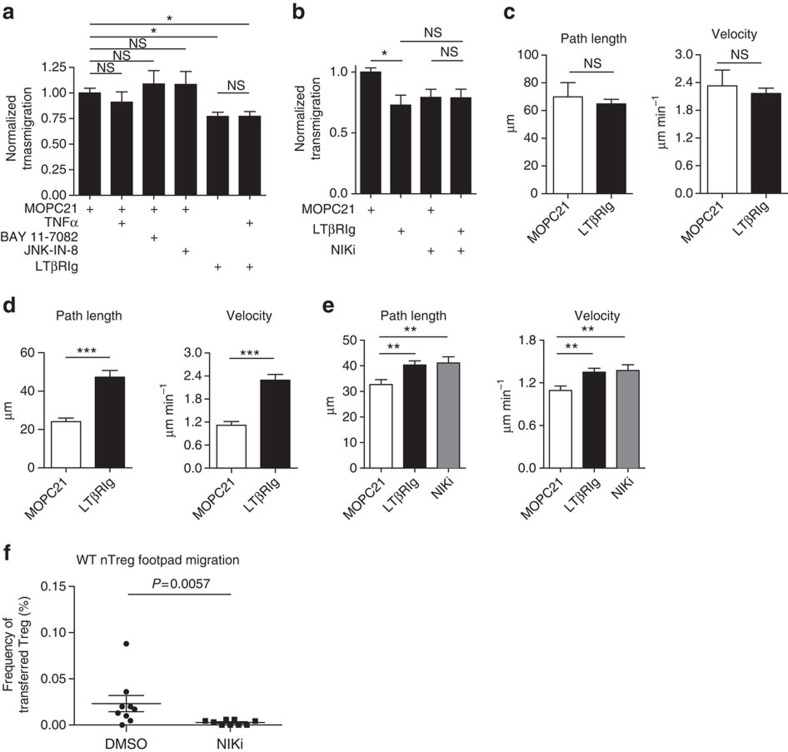
Treg migration through and two-dimensional interactions with LEC depend on LTβR-dependent non-canonical NFκB signalling in LEC. (**a**,**b**) Aza Treg migrated across iSVEC4-10 to CCL19 in the presence of LTβRIg or control MOPC21. iSVEC4-10 pretreated as indicated. Migration normalized to MOPC21. Six transwells per condition from two experiments in **a** and nine transwells per condition from three experiments in **b**. (**c**,**d**,**e**) Aza Treg two-dimensional movement on iSVEC4-10 10 min (**c**) or 3 h (**d**,**e**) after Treg addition in the presence of CCL19 gradient. Images collected once per minute for 30 min. A total of 78 tracked cells MOPC21, 117 LTβRIg from 1 experiment representative of 4 in **c**; 100 tracked cells MOPC21, 87 LTβRIg from 1 experiment representative of 2 in **d**; 155 tracked cells MOPC21, 151 LTβRIg, 118 NIKi from 1 experiment representative of 2 in **e**. (**f**) Resting WT C57BL/6J CD25+CD4+ nTreg enriched by magnetic beads, labelled with CFSE, mixed with DMSO or NIKi in DMSO and injected into footpads. Popliteal LN procured 12 h later and analysed by flow cytometry. Nine mice for each treatment from two experiments in **f**, *P<0.05, **P<0.01, ***P<0.001 by Bonferroni post test of one-way analysis of variance in **a**,**b** and **e**, unpaired two-tailed Student's *t*-tests in **c** and **d** and Mann–Whitney in **f**. Error bars are s.e.m. NS, not significant.

LTβR stimulation activates three pathways: c-Jun N-terminal kinase (JNK); classical nuclear factor kappa-light-chain-enhancer of activated B cells (NFκB); and non-canonical NFκB^27^. To determine which pathway regulated transendothelial migration, Tregs were migrated to CCL19 across iSVEC4-10 layers treated with the JNK inhibitor JNK-IN-8 or the classical NFκB inhibitor BAY 11-7082. These treatments did not decrease Treg transmigration (Fig. 5a). To further confirm the lack of effect of the JNK and classical NFκB pathways on Treg transmigration, iSVEC4-10 were treated with TNFα, which only induces signals via those pathways. While SVEC4-10 have functional TNFα receptors, which permit them to increase expression of cell surface adhesion molecules^26^, TNFα failed to increase Treg transmigration or reverse LTβRIg-induced inhibition (Fig. 5a). In contrast, pretreatment of iSVEC4-10 cell layers with 4H-isoquinoline-1,3-dione (NIKi), an inhibitor of the non-canonical NFκB-inducing kinase (NIK)^28^,^29^, inhibited Treg transmigration. NIKi inhibition was not additive with LTβRIg, suggesting both blocked the same pathway (Fig. 5b).

Since LTβRIg impaired Treg transmigration across lymphatic endothelium, we hypothesized that it altered interactions of Treg with lymphatic endothelium before or during transmigration. To determine how LT engagement regulated migration, Treg movement across iSVEC4-10 layers was assessed by live imaging. If imaging began 10 min after Treg addition, there were no differences in distances travelled or velocities between LTβRIg-treated and control groups (Fig. 5c). However, if imaging was initiated 3 h after Treg addition, LTβRIg resulted in increased Treg distances and velocities compared with controls (Fig. 5d), suggesting time- and LT-dependent Treg–LEC interactions resulting in Treg slowing, adhesion and transmigration. These findings are consistent with the ear live imaging data (Fig. 3l). NIKi pretreatment, like LTβRIg, also led to increased distances and velocities of Treg movement, again suggesting that both agents blocked the same pathway (Fig. 5e). We also co-injected NIKi at 10 mg kg^−1^ into footpads with WT nTreg and found that NIKi inhibited Treg migration to draining LN (Fig. 5f). These findings support a role for NIK controlling Treg interstitial and lymphatic migration *in vivo* and *in vitro*.

### Tregs induce protrusion on the basal surface of LEC

**Figure 6:**
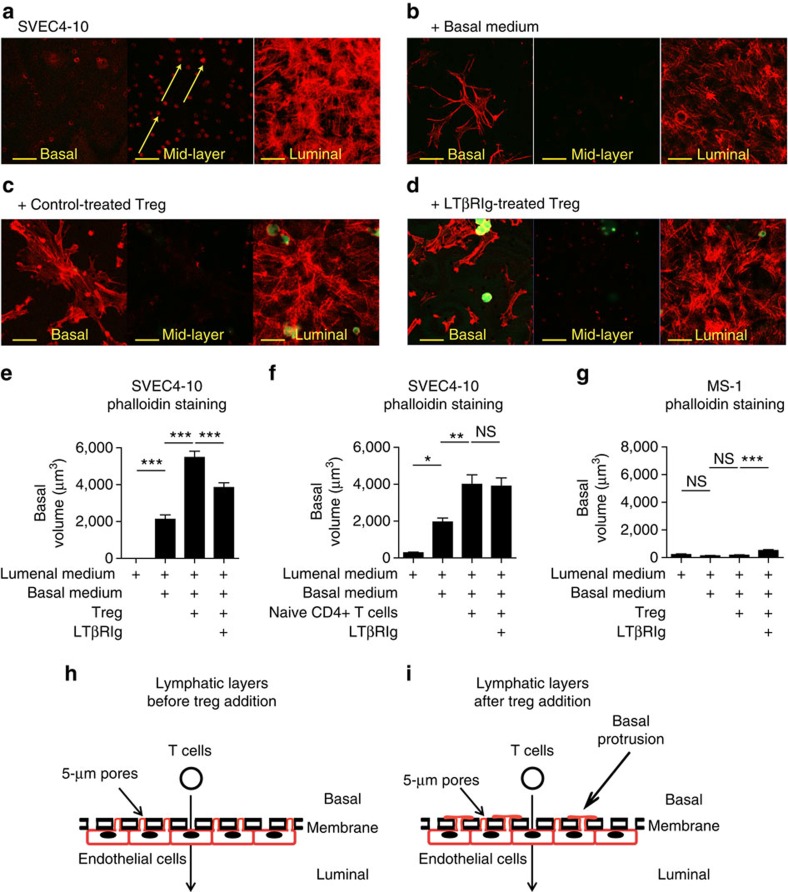
Tregs induce lamellipodia-like structures on the basal surface of lymphatic endothelium. (**a**–**d**) Representative images of Aza Treg in iSVEC4-10 layers. CFSE+ Treg in green; phalloidin in red; × 63. Yellow scale bar, 28 μm. Yellow arrows indicate thin f-actin+ protrusions invading the pores of the membrane and approaching its basal surface as described in the text. (**e**) Summary of all fields in **a**–**d** showing volume of phalloidin+CFSE negative structures per field in μm^3^. (**f**) Same as **e** but with naive non-Treg CD4 T cells. (**g**) Same as **e** with iMS-1 instead of iSVEC4-10. (**e**–**g**) A total of 20–40 fields per condition from 1 experiment representative of 2–3 experiments. (**h**,**i**) Cartoons depicting endothelial protrusion growth displayed in *xz* plane view. *P<0.05, **P<0.01, ***P<0.001 by Bonferroni's post test of one-way analysis of variance. Error bars are s.e.m. NS, not significant.

Leukocytes interact dynamically and intimately with endothelial cells during transmigration, engaging multiple receptors and ligands and involving downstream cytoskeletal changes. To probe Treg–SVEC4-10 interactions over time, iSVEC4-10 layers were stained with phalloidin to reveal filamentous actin (f-actin). iSVEC4-10 grown for 2–3 days on transwell inserts formed confluent monolayers with intense f-actin staining on the lower or luminal surface, and thin f-actin+ protrusions invading the pores of the membrane and approaching its upper/basal surface (Fig. 6a, arrows, and 6h). If iSVEC4-10 were incubated for an additional 4 h after addition of medium to the basal and luminal sides, flat f-actin+ structures with protrusions resembling lamellipodia spread on the basal surfaces (Fig. 6b,e,i). When measuring these protrusions, we measured the volume of all phalloidin+ (f-actin+) structures on the basal side of the plastic membrane. At the start of the experiment, there were no such structures, as there had only been medium on the luminal but not the basal side of the membrane during initial cell growth. These protrusions did not universally resemble lamellipodia, but in the control MOPC21-Treg condition most of the structures that spread from the pores took a broad, flat, sheet-like form consistent with lamellipodia. These protrusions were enhanced and more prominent after MOPC21-treated Treg were added to the basal surface (Fig. 6c,e). If Tregs were pretreated with LTβRIg growth of the basal structures was significantly reduced (Fig. 6d,e). In contrast, naive non-Treg CD4+ T cells induced less growth of these structures, and that growth was insensitive to LT blockade (Fig. 6f). In contrast to iSVEC4-10, iMS-1 layers failed to grow basal structures, exhibiting growth of only a few rudimentary cytoplasmic protrusions (Fig. 6g).

### Basal LEC protrusion growth is regulated by NIK

**Figure 7:**
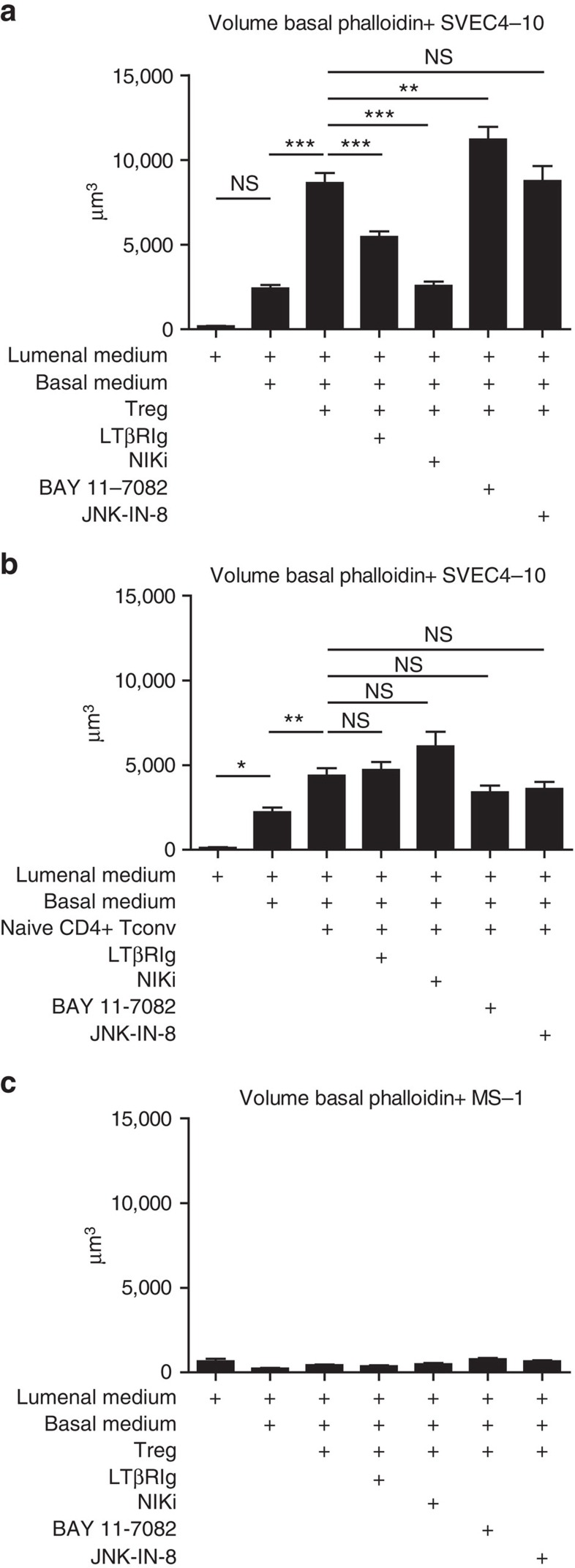
Treg modulation of iSVEC4-10 basal protrusions is mediated by signals via NIK. (**a**–**c**) Summary volumes of phalloidin+CFSE negative structures per × 63 field 4 h after addition of Aza Treg (**a**,**c**) or naive non-Treg CD4+ T cells (**b**) to iSVEC4-10 (**a**,**b**) or iMS-1 (**c**). A total of 20–40 fields per condition from 1 experiment representative of 2–3 experiments. *P<0.05, **P<0.01, ***P<0.001 by Bonferroni post test of one-way ANOVA. Error bars are s.e.m. NS, not significant.

We hypothesized that the basal structures induced in SVEC4-10 by Treg were involved in promoting Treg transmigration and that their growth would be susceptible to inhibition by the same treatments that prevented Treg transmigration. Specifically, inhibition of NIK would reduce growth, while inhibition of JNK or classical NFκB would not. SVEC4-10 pretreated with NIKi demonstrated significantly less growth of basal structures compared with controls (Fig. 7a). In contrast, pretreatment of iSVEC4-10 layers with inhibitors of JNK or classical NFκB (JNK-IN-8 and BAY 11-7082, respectively), did not alter the growth of basal f-actin+ structures and in the case of BAY 11-7082, even increased them slightly (Fig. 7a). In agreement with the previous data, the growth of basal structures after the addition of naive non-Treg CD4+ T cells was substantially less than that induced by Treg, and was not modulated by any of the pharmacological inhibitors (Fig. 7b). Treg did not induce these basal structures when added to inverted MS-1 layers (Fig. 7c) and the pharmacological inhibitors did not alter MS-1 growth. Furthermore, primary LEC basal protrusion growth in response to Treg could be also inhibited by LT blockade or NIKi treatment (Supplementary Fig. 5a). Thus, signals from the LTβR were transduced in SVEC4-10 cells through the non-canonical NFκB pathway via NIK. Induction of this pathway was particular to Treg and did not occur with the BEC cell line MS-1.

The SVEC4-10 basal structures induced by Treg were further characterized by confocal microscopy. Treg were in contact with basal lymphatic protrusions of SVEC4-10 and mouse primary LEC (Supplementary Fig. 5b,c), VCAM-1 was observed in SVEC4-10 basal protrusions near Treg (Supplementary Fig. 5b,c, green arrows), and LTβRIg and NIKi both decreased the mean fluorescence intensity of VCAM-1 close to the Treg (Supplementary Fig. 5d). VCAM-1 intensity in the vicinity of CD4+ non-Treg did not change with LTβRIg treatment (Supplementary Fig. 6a). There was no evidence that CCL19 was concentrated in or presented by basal protrusions and no change was observed in CCL19 staining intensity in the vicinity of Treg throughout the lymphatic layers with either LTβRIg or NIKi treatment (Supplementary Fig. 6b). These findings are consistent with a model in which Treg LTα1β2 engagement with lymphatic LTβR recruits or induces VCAM-1. Treg responded by decreasing distance and velocity, probably as a result of adhesive interactions, and migrating through the endothelial layer. LEC responded by extending structures that may engage the Treg and by altering the distribution of VCAM-1 with respect to Treg. Thus, there was an active and bidirectional interaction between Treg and LEC.

### LT-dependent Treg migration across LEC depends on VCAM-1

**Figure 8:**
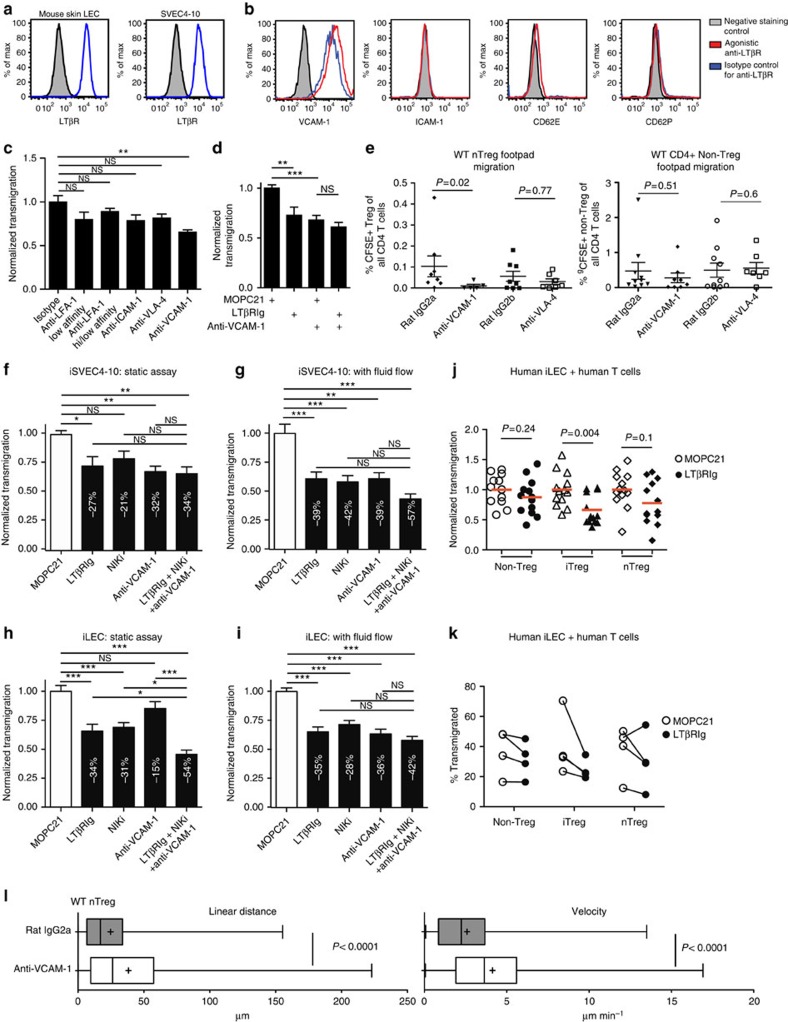
Treg transmigration through lymphatic endothelium depends on VCAM-1 *in vitro* and *in vivo*. (**a**) SVEC4-10 or mouse skin LEC: grey histograms, isotype control; blue: LTβR. Representative of 3 experiments. (**b**) SVEC4-10 cultured 48 h, with or without agonistic 1 μg ml^−1^ anti-LTβR last 24 h, stained for indicated molecules. Isotype control, black histograms; indicated antibody, blue (control-treated) and red (anti-LTβR-treated). Representative of two experiments. (**c**,**d**) Aza Treg migrated across iSVEC4-10 to CCL19. SVEC4-10 (anti-ICAM-1 and anti-VCAM-1) or Treg (anti-LFA-1, anti-VLA-4, MOPC21 and LTβRIg) pretreated as indicated. Migration normalized to control. Six transwells from two experiments in **c** and nine transwells from three experiments in **d**. (**e**) Footpad migration. CFSE-labelled WT nTreg or naive CD4+ non-Treg co-injected with Rat IgG2a or anti-VCAM-1 or pretreated with Rat IgG2b or anti-VLA-4. Per cent CFSE+ cells of popliteal LN CD4 T cells shown. Treg: 5–8 mice from 3 experiments; non-Treg: 7–10 mice from 3 experiments. (**f**–**i**) Aza Treg transwell migration across iSVEC4-10 (**f**,**g**) and primary mouse iLEC (**h**,**i**). Under static conditions (**f**,**h**) and with fluid flow (**g**,**i**). T cells (MOPC21 and LTβRIg) or endothelial cells (NIKi and anti-VCAM-1) treated as indicated. White numerals indicate % decline compared with MOPC21. Migration normalized to MOPC21. Results from nine transwells per condition from three experiments in **f** and **g**, and eight transwells from two experiments in **h** and **i**. (**j**,**k**) Transwell migration of human T cells across human skin iLEC to murine CCL19. T cells treated with MOPC21 or murine LTβRIg. (**j**) Results by well, 13 per condition from 4 experiments, red bars denote mean. (**k**) Results by human T-cell donor, control paired with LTβRIg. (**l**) Summary of Treg movement in ears during 30 min of image acquisition. In all, 563 Rat IgG2a- and 511 anti-VCAM-1-treated cells tracked using Volocity 6.1.1. Results from 2–3 ears per condition, 1 × 20 field per ear, from 2 experiments. Box and whiskers plot showing minimum, maximum, mean (+), median (bar), 25th and 75th percentiles. *P<0.05, **P<0.01, ***P<0.001 by Bonferroni post test of one-way analysis of variance (**c**,**d**,**f**–**i**), unpaired Student's *t*-test (**j**,**l**) and Mann–Whitney non-parametric test (**e**). Error bars are s.e.m in **c**–**g**,**h**,**i**. NS, not significant.

To better understand Treg–LEC interactions, surface molecules expressed by SVEC4-10 were characterized, and both primary mouse skin LEC and SVEC4-10 expressed high levels of LTβR (Fig. 8a). In agreement with our previous work^26^, SVEC4-10 expressed substantial VCAM-1, but very little ICAM-1, CD62E or CD62P in the steady state (Fig. 8b). Stimulation of SVEC4-10 with the agonistic anti-LTβR mAb AF.H6 induced a slight increase in VCAM-1 surface expression but did not alter expression of ICAM-1, CD62E or CD62P (Fig. 8b). In keeping with these expression patterns only VCAM-1 blockade reduced Treg transmigration (Fig. 8c). VCAM-1 is a known target of LTβR signalling^30^. Combined treatment with anti-VCAM-1 mAb plus LTβRIg was not additive (Fig. 8d), suggesting both mediated migration inhibition via a common pathway. In keeping with the *in vitro* results, VCAM-1 antibody co-injection prevented Treg but not CD4+ non-Treg migration from footpad to draining LN while anti-VLA-4 did not inhibit migration of either Treg or CD4+ non-Treg (Fig. 8e). We developed a simple model of fluid flow from the upper to the lower well to more faithfully replicate the *in vivo* situation. This was accomplished by filling the upper transwell as full as possible while reducing the volume in the lower transwell to the minimum required to cover the bottom of the transwell insert. This resulted in an initial pressure head of ∼0.8–0.9 cm H_2_O that declined during the experiment. This is consistent with estimates of *in vivo* interstitial pressure gradients from 0.2 to 0.8 cm H_2_O (ref. 31). Using this system, LTβRIg, NIKi and anti-VCAM-1 often resulted in greater Treg migration inhibition under flow than in static assays across both iSVEC4-10 (Fig. 8f,g) and primary mouse iLEC (Fig. 8h,i). This may explain in part why the *in vivo* assays showed greater migration inhibition than did the static *in vitro* assays. Additional *in vitro* migration assays were performed using human T cells and primary human LEC with fluid flow. Mouse LTβRIg was used since it binds human LTα1β2 with an affinity comparable to human LTβRIg (ref. 32). Using four unique donors, LTβRIg inhibited migration of anti-CD3-stimulated human iTreg but not non-Treg CD4 T cells (Fig. 8j). Stimulated human nTreg tended to migrate less when treated with LTβRIg (Fig. 8j). Three of four nTreg donors showed a reduction in migration when treated with LTβRIg and if the fourth donor were excluded the *P* value would change from *P*=0.1 to 0.03, however such exclusion is not easily justifiable (Fig. 8j,k). Given the inherent variability in T-cell samples from different human donors and the possibility of alloreactivity to the LEC also varying by donor, such variability by donor was not surprising. Finally, we again tracked the movement of live WT nTreg in ear explants. Treg in the vicinity of lymphatics moved farther and faster when co-injected with anti-VCAM-1 but not when co-injected with control Rat IgG2a (Fig. 8l and Supplementary Movies 5 and 6), consistent with the results using LTβRIg (Fig. 3l and Supplementary Movies 1 and 2). In summary Treg depend on LT-LTβR and VCAM-1 interactions with lymphatic endothelium for translymphatic migration under static conditions, and under flow, both *in vitro* and *in vivo*, in both mice and humans.

## Discussion

We showed here that among the T-cell subsets examined, Tregs preferentially express and use LTαβ for migration across lymphatic endothelium *in vitro* and into afferent lymphatics and migration to LN *in vivo*. To our knowledge this is the first demonstration of T-cell LTαβ playing a direct role in T-cell migration. The finding that LTαβ is acutely involved in T-cell migration, particularly Treg, is important and may require reinterpretation of results in which LT receptors or ligands were manipulated. The preferential use of LTαβ by Treg for migration, but not by non-Treg T cells, also suggests that LTαβ/LTβR interactions may be targets for selectively modulating Treg migration. However, activated helper T cells, Th1 and Th17 cells in particular, have also been described to express LTαβ(ref. 19), and may use this for migration in other models or circumstances. Further, T-cell LTαβ has other functions aside from enhancing lymphatic transmigration, for example, enhancing dendritic cell maturation and function^33^, mast cell activation^34^ and maintaining fibroblastic reticular cell structure and function^35^. It remains to be seen what other functions Treg- and non-Treg-expressed LT might exert. It is also not clear why other cell types, such as naive T cells, do not require LT for lymphatic migration. The answer may have to do with the combination, amount and activation state of integrins and other adhesion molecules, as well as chemokine receptors, that Treg express compared with non-Treg T cells. Treg lymphatic transmigration also depended at least in part on VCAM-1, and blocking data suggested that the roles of LTαβ/LTβR and VCAM-1 in transmigration were part of the same pathway. Interestingly, Treg transmigration did not depend on α4 integrins (VLA-4), which are major binding partners for VCAM-1. However, there is evidence that α4 is dispensable for T-cell transmigration across HEV^36^, and that integrins are not required for lymphatic transmigration of DC^11^, although VCAM-1 is important for DC transmigration across inflamed lymphatic endothelium^37^. There are integrin ligands for VCAM-1 aside from α4 integrins^38^,^39^, and non-integrin ligands for VCAM-1 can also regulate transendothelial migration^40^. Thus, there is precedent for VCAM-1 playing an α4-independent role in lymphatic transmigration. This mechanism also depended on NIK and non-canonical NFκB signalling. While VCAM-1 is typically thought to be regulated via classical NFκB signalling, embryonic fibroblasts from aly/aly mice, which express a mutated NIK, make VCAM-1 mRNA but fail to express surface VCAM-1 in response to LTβR stimulation, suggesting that NIK regulates VCAM-1 post-translationally^41^,^42^. VCAM-1 protein expression has also been shown to be NIK-dependent in response to other stimuli^43^.

Tregs are among the most substantial populations of T cells to migrate to LN via afferent lymphatics^5^, and this process is required for optimal Treg-mediated protection of islet allografts^2^. Therefore, this step could be an important checkpoint in the regulation of inflammatory responses in non-lymphoid tissues. Treg proportional representation in inflamed skin is less than their representation among cells that reach the draining LN^5^. This suggests Treg may gain a migratory advantage by inducing increased expression by LEC of molecules conducive to transmigration, or by inducing LEC to engage with and physically promote the movement of Treg across or through lymphatic endothelium. Treg may also impair migration of other cells through lymphatic endothelium^2^.

LEC and BEC responded differently to LTβR engagement by Treg LTαβ. Only Treg migration across LEC was impeded by LTβRIg, and only LEC extended basal protrusions in response to engagement of LTβR by Treg LT. Human umbilical vein endothelial cells, BEC and human dermal microvascular endothelial cells, a mixture of LEC and BEC, have been reported to extend microvillus-like projections or ‘docking structures' from their apical surfaces around infiltrating lymphocytes^44^,^45^,^46^. Others have found similar structures surrounding human DCs interacting with the apical face of LEC^47^. These structures are microvillus-like projections one to several microns long that form symmetrical rings around transmigrating cells, while the protrusions we observed were tens of microns in width and not restricted to encircling the base of transmigrating Treg. In contrast to these studies^44^,^45^,^46^,^47^, we examined interactions of T cells with the basal side. Together these findings all support a model where endothelial cells play an active role in transendothelial migration by engaging leukocyte via surface receptors and/or other physical engagement.

Tissue to lymphatic migration requires that cells crawl through a three-dimensional interstitial matrix, find terminal lymphatic capillaries with assistance from fluid flow and chemokine gradients, and traverse a minimal basement membrane. LTβRIg treatment resulted in larger average distances of Treg from lymphatics in ear pinnae. This suggests LTβRIg treatment might have resulted in impaired generation of chemokine gradients. The fibroblasts that produce the interstitial matrix express LTβR so it is possible that LT-dependent interactions with tissue fibroblasts were also required for efficient interstitial migration of Treg. Our data suggest that LTαβ/LTβR interactions may regulate interstitial migration in addition to regulating transmigration across lymphatic endothelium, revealing another unexpected function of the LTαβ/LTβR axis.

## Methods

### Animals

C57BL/6 (WT, *Ltα*^−/−^, *Ltbr*^−/−^) and BALB/c mice (8–14 weeks) were purchased from The Jackson Laboratory (Bar Harbor, ME). Foxp3GFP^48^ mice on a C57BL/6 background were from Dr A. Rudensky (Memorial Sloan Kettering Cancer Center). All experiments were performed with age- and sex-matched mice.

### Mouse T-cell purification and culture

Mouse LN and spleens were passed through 70-μm nylon mesh (Fisher Scientific) to produce single-cell suspensions and were enriched for CD4+ T cells using CD4+ negative selection (Stemcell Technologies). Cells were stained with antibodies to CD4, CD25 and CD44 as detailed in ‘Flow cytometry section. CD4^+^CD44^lo^CD25^−^ *Ltα*^−/−^ cells, CD4^+^CD44^lo^CD25^−^Foxp3GFP^−^ WT cells and CD4^+^CD25^+^ *Ltα*^−/−^ cells and CD4^+^CD25^+^Foxp3GFP^+^ WT cells were sorted using a FACS Aria I (BD Biosciences). Cell purity was >98%. T cell-depleted, 800 rad-irradiated C57BL/6 splenocytes were used as stimulator cells. Purified CD4^+^CD44^lo^CD25^−^ (or CD4^+^CD44^lo^CD25^−^Foxp3GFP^−^) T cells (5 × 10^4^ cells per well) were cultured with stimulator cells (5 × 10^4^ cells per well) with IL-2 (20 ng ml^−1^, eBioscience), anti-CD3ɛ mAb (1 μg ml^−1^, clone 145-2C11, eBioscience, 16-0031) for T_eff_, with the addition of human TGF-β1 (10 ng ml^−1^, eBioscience) for iTreg, and TGF-β1 and 5-Aza-2′-deoxycytidine (10 μM, Sigma-Aldrich) for Aza Treg, in a final volume of 200 μl of RPMI 1640 supplemented with 10% FBS (Gemini), 1 mM sodium pyruvate (Lonza), 2 mM L-glutamine (Lonza), 100 IU ml^−1^ penicillin (Lonza), 100 μg ml^−1^ streptomycin (Lonza), 1 × non-essential amino acids (Lonza) and 2 × 10^−5^ M 2-ME (Sigma-Aldrich) in U-bottom 96-well plates (Corning). Cells were cultured for 5 days at 37 °C in 5% CO_2_. Aza Treg culture media replaced at 24 h with medium containing anti-CD3ɛ, IL-2 and TGF-β1. All were given fresh medium with appropriate cytokines but no anti-CD3ɛ mAb at 72 h. CD8 T cells were enriched by magnetic bead negative selection (Stemcell Technologies) then labelled with CFSE for footpad studies, or no CFSE for *in vitro* migration studies. CD44^hi^CD4+ T cells were enriched for CD4+ cells by magnetic bead negative selection (Stemcell Technologies), and CD44^hi^ T cells were further purified using anti-CD44-PE (1:1,000, clone IM7, eBioscience, 12-0441), anti-PE magnetic microbeads and magnetic separation (Stemcell Technologies).

### *In vitro* suppression assays

CD4+CD25+ nTreg and CD4+CD25− non-Treg T cells were sorted from indicated strains. Treg were labelled with CFSE (Life Technologies) and responder non-Treg with eFluor 670 (Molecular Probes, Eugene, OR). Responder CD4+CD25− T cells (5 × 10^4^ cells per well) were cultured with or without Treg at responder:Treg ratios of 1:0, 1:1, 2:1, 4:1 and 8:1 with irradiated (800 rad) syngeneic T cell-depleted splenocytes (5 × 10^4^ cells per well) in 96-well U-bottom plates (Corning) and anti-CD3ɛ mAb (1 μg ml^−1^, clone 145-2C11, eBioscience, 16-0031) for 3 days. Cells were collected, and cell division was measured by assessing relative eFluor 670 dilution on an LSRFortessa cytometer (BD).

### Cell lines

SVEC4-10 (CRL-2181) and MS-1 (CRL2279) cells were from American Type Culture Collection. Cell lines were expanded after arrival from American Type Culture Collection, verified as mycoplasma-free, were aliquoted and frozen and used until passage 10 after thawing. SVEC4-10 and MS-1 were cultured in DMEM with 4.5 g l^−1^ glucose (Lonza), containing 10% (v/v) FBS (Benchmark, Gemini), 2 mM L-glutamine (Lonza), 100 IU ml^−1^ penicillin (Lonza) and 100 μg ml^−1^ streptomycin (Lonza) in 75-cm^2^ vented culture flasks (Corning) in a horizontal orientation until they reached 70–85% confluence, then treated with 0.05% Trypsin-EDTA (Gibco) for 5–7 min at 37 °C for detachment, and passaged to new flasks at a dilution of 1:6. Cultures were used up to passage five.

### Primary LEC

C57BL/6 mouse primary dermal LECs (C57-6064 L) and human primary dermal LECs (H-6064 L) were from Cell Biologics, Inc. (Chicago, IL), and were cultured according to the manufacturer's instructions in manufacturer-provided mouse endothelial cell medium supplemented with 5% FBS, 2 mM L-glutamine, 100 IU ml^−1^ penicillin, vascular endothelial growth factor, endothelial cell growth supplement, heparin, epidermal growth factor, hydrocortisone or human endothelial cell medium with 10% FBS, 2% endothelial cell supplement, 2 mM l-glutamine, 100 IU ml^−1^ penicillin, vascular endothelial growth factor, heparin, epidermal growth factor, fibroblast growth factor and hydrocortisone. To split cells, they were incubated with 3.0 ml warm (37 °C) 0.25% Trypsin-EDTA solution (Gibco) for 3–5 min and quenched with 8–10 ml of Cell Biologics' Cell Culture Medium supplemented with 5–10% FBS as soon as cells had detached. Cells were plated at a 1:2 ratio in fresh flasks pre-coated with Gelatin-Based Coating Solution (Cell Biologics) in a humidified, 5% CO_2_ incubator at 37 °C. Culture media was changed the following day and every 24–48 h thereafter. LECs were grown on transwell inserts as described for SVEC4-10, but were seeded at 2 × 10^5^ cells per insert and grown 2 days before use. Inserts for human LEC were coated with 0.2% gelatin, but mouse LEC required coating for 2 h at 37 °C with 10 μg recombinant human laminin 511 (Biolamina AB, Sundbyberg, Sweden) in 100 μl Dulbecco's phosphate-buffered saline to make confluent monolayers.

### Human T-cell purification and culture

Naive CD4+ T cells (CD4+25−127+CD45RA+) were sorted from non-mobilized peripheral blood apheresis products (Memorial Blood Center, St. Paul, MN, USA) using a FACSAria as in ref. 49. These cells were used to produce ‘effectors' and induced Treg (iTreg). Both were stimulated with a K562 cell line engineered to express CD86 and the high-affinity Fc Receptor (CD64) (KT86/64)^49^. KT86/64 were incubated with anti-CD3 (OKT3, Miltenyi, #170-076-116) at 1 μg per 1 × 10^6^ KT cells per ml for 10 min, then were washed, irradiated with 10,000 cGray and frozen. The KT86/64 cells were thawed and washed directly before incubation with T cells. OKT3-incubated KT86/64 cells were cultured with T cells at a 2:1 Tcell:KT86/64 ratio in the presence of IL-2 (300 IU ml^−1^, Novartis). iTregs were expanded in the presence of TGF-β1 (10 ng ml^−1^, R&D) and rapamycin (109 nM, Rapammune, Wyeth-Ayerst), while control ‘effector' cells were cultured without TGF-β1 and rapamycin. nTregs were purified from fresh umbilical cord blood units within 72 h of isolation from the donor (National Placental Blood Program, New York Blood Center) by positive selection using directly conjugated anti-CD25 PE magnetic microbeads (CliniMACS CD25 beads, Miltenyi, #274-01) with AutoMACS or CliniMACS cell separators (Miltenyi, Posseld2 program). CD4CD25^+^ cells (average 64% CD4^+^) were cultured with KT86/64 cell lines at a Treg to KT cell ratio of 2:1 (ref. 30). CD25^+^ cells were cultured in X-Vivo-15 (BioWhittaker, Walkersville, MD) or RPMI 1640 (Invitrogen, Carlsbad, CA) media supplemented with 10% human AB serum (Valley Biomedical, Winchester, VA), L-glutamine (Invitrogen) and *N*-acetylcysteine (American Regent, Shirley, NY). Recombinant IL-2 (300 IU ml^−1^, Novartis) was added on day 3 and maintained for culture duration. Cells were cultured for 18–21 days and split every 2–3 days. nTregs were consistently >85% CD127^−^Foxp3^+^ and ∼70% Foxp3^+^Helios^+^. iTregs were consistently 60–70% CD127^−^Foxp3^+^ and ∼10% Foxp3^+^Helios^+^. Effectors were consistently ∼10% CD127^-^Foxp3^+^ with few to no Foxp3^+^Helios^+^ cells.

### Blocking antibodies for *in vitro* studies

Functional grade-blocking anti-VCAM-1 (clone 429, eBioscience, 16-1061), ICAM-1 (clone YN1, eBioscience, 160541), VLA4/α4 integrin (clone R1-2, eBioscience, 16-0492), high+low-affinity LFA-1/CD11a (clone M17/4, eBioscience, 16-0111) and low-affinity CD11a (clone 2D7, BD, 553118), Rat IgG2a (clone eBR2a, eBioscience, 16-4321) and Rat IgG2b (clone eB149/10H5, eBioscience, 16-4031) were used to treat endothelial layers or T cells at 4 °C for 30 min at 10 μg ml^−1^. In some experiments, low-endotoxin, no-azide (LEAF) grade anti-IL-10 (clone JES5-2A5, Biolegend, 504903), LEAF grade anti-TGF-β1 (clone 19D8, Biolegend, 521703) or LEAF Grade Rat IgG1k (RTK2071, Biolegend, 400413) were incubated with T cells at 10 μg ml^−1^ for 30 min at 4 °C before transmigration. T cells were washed 2 × before use except in the case of anti-TGF-β1, anti-IL-10 and control Rat IgG1k, while VCAM-1 and ICAM-1 blockades of endothelial cells were also maintained during assays.

### Small-molecule inhibitors and other treatments

Small-molecule inhibitors were used to treat endothelial cell layers. JNK 1 and 2 were blocked with JNK-IN-8 (Calbiochem) at 2 μM for 3 h at 37 °C (ref. 50), the classical NFκB pathway was blocked with BAY-11-7082 (Sigma) at 20 μM for 1 h at 37 °C (ref. 51), and NIK was blocked with NIKi (Enamine) at 50 μM for 2 h at 37 °C (refs 28, 29). Cell layers were washed 3 × with IMDM 1 × insulin/transferrin/selenium (ITS; Invitrogen) containing 0.5% (w/v) fatty acid-free BSA (Gemini) before use. For *in vivo* use NIKi was prepared at a stock concentration of 500 mM in dimethylsulphoxide, diluted in PBS/T cell mixtures to a final volume of 20 μl and injected into the hind footpad at 10 mg kg^−1^. Murine IL-10 and TGF-β1 were from EBiosciences. For transwell migration in the presence of these cytokines, 10 ng ml^−1^ each or both together was added to T-cell preparations at the start of the assay.

### Transendothelial migration

In all, 1.5 × 10^5^ MS-1 or SVEC4-10 in 100 μl DMEM High Glucose (Lonza) 10% FCS (Gemini) on the upper surface of a transwell insert previously coated with 0.2% (w/v) gelatin (Bio-Rad) for 2 h at 37 °C. Alternatively, inserts were flipped 180° and cells seeded on lower surface (inverted culture). After 2 days, cell confluency was assessed with a quick haematoxylin and eosin stain (VWR). In some experiments, endothelial cells were pretreated with antibodies, or JNK or NFκB inhibitors and then washed three times with IMDM 1 × ITS containing 0.5% (w/v) fatty acid-free BSA (Gemini), and migration across cell layers was assessed. T-cell subsets were prepared in IMDM+1 × ITS containing 0.5% (w/v) fatty acid-free BSA and incubated for 30 min at 37 °C with 50 μg ml^−1^ of LTβRIg (gift from Biogen Idec) or control MOPC21 (BioExCell). A total of 1–3 × 10^5^ cells in 100 μl were added to the upper wells of a 24-well transwell plate with polycarbonate membrane inserts and pore sizes of 5 μm (Corning International). Lower wells contained CCL19 (0.5 μg ml^−1^), CCL5 (0.5 μg ml^−1^), S1P (100 nM), in 600 μl IMDM 1 × ITS plus 0.5% (w/v) fatty acid-free BSA. T cells that migrated to the lower well after 4 h at 37 °C were counted in four to eight full 1-mm corner squares with a haemocytometer. Migration with fluid flow was performed as described for static assays with the following modification: upper wells were filled with 340 μl medium and the lower wells with 360 μl. This resulted in an initial static pressure head of ∼0.8–0.9 mm H_2_O.

### Time-lapse microscopy

CFSE+ T cells (1 × 10^4^ cells per transwell) migrating across endothelial monolayers to CCL19 (0.5 μg ml^−1^) were visualized by fluorescence microscopy (Axiovert 200M) with a × 20 objective. One image was captured every minute for 30 min with Axiovision 4.7.1 software (Carl Zeiss Jena GmbH). Mean distance from origin and mean velocity analysed with Volocity version 6.1.1 software (Perkin Elmer).

### Conventional and confocal microscopy

CFSE-stained T cells (5 × 10^4^ cells per transwell) migrated for 4 h across endothelial monolayers to CCL19 (0.5 μg ml^−1^), fixed for 20 min at 4 °C with 4% (w/v) paraformaldehyde (Affymetrix), and paraformaldehyde then neutralized for 10 min at 4 °C with PBS 0.1 M glycine (Sigma-Aldrich), pH 7.4. Cells were permeablized with PBS 0.2% (v/v) Triton X-100 (Sigma-Aldrich), stained for 30 min at 4 °C with Alexa fluor 555-phalloidin (Life Technologies) and washed with PBS containing 0.2% (v/v) Triton X-100 followed by PBS alone. In some experiments cell layers were also stained with polyclonal goat anti-VCAM-1 at 2 μg ml^−1^ (sc-1504, Santa Cruz) and detected with Donkey anti-goat Alexa Fluor 647 at 5 μg ml^−1^ (Jackson Immunoresearch, 705-606-147). Transwell membranes were transferred onto glass slides and visualized by confocal microscopy (Zeiss LSM 510 Meta, and LSM5 Duo) with a × 63 or × 40 objective, respectively. *Z*-stack images were acquired every 1 μm with 2-μm slice thicknesses for examination of *in vitro* endothelial monolayers. Images were analysed with Volocity version 6.1.1 software (Perkin Elmer).

### *Ex vivo* live imaging

C57BL/6 recipient ears were depilated with Nair Lotion Hair Removal for 1 min, and loose hair and depilatory solution were washed with several saline rinses. Three days later mice were anaesthetized with ketamine/xylazine and given 1 μg azide-free Alexa-488-labelled anti-LYVE-1 in 10 μl PBS (clone ALY7, eBioscience, Custom Product) into the ear pinna to identify lymphatic vessels as previously shown^52^. Six hours later, 1 × 10^6^ magnetic bead-purified efluor 670+ (eBioscience), CD25+ nTreg derived from 5–7 C57BL/6 donor LN and spleens or 6–8 *Ltα*^−/−^ donor spleens per experiment were injected into the ear pinna in 10 μl. Treg were treated with LTβRIg or MOPC21 as indicated for 30 min at 37 °C and then washed 2 × with PBS. In some experiments 5 μg of no-azide, low-endotoxin (NA/LE) grade anti-VCAM-1 (429, BD) or NA/LE Rat IgG2a isotype control (BD) were co-injected with T cells. Four hours after T-cell injection mice were killed, ears removed, secured to a Sylgard 184 (Dow Corning) silicone block with surgical tape and covered with HEPES buffered saline at 37 °C for imaging. Movies were recorded beginning no later than 30 min after ear removal. *Z*-stacks (3-μm slices, 3-μm interval, 18–24-μm stack height) were collected once per minute for 30 min with a Zeiss 710 upright microscope in confocal mode using a × 20 water immersion objective. Movies were analysed with Volocity version 6.1.1 software (Perkin Elmer).

### Flow cytometry

Mouse LN and spleen were passed through 70-μm nylon mesh screens (Fisher Scientific), to produce single-cell suspensions. Cell suspensions were treated with anti-CD16/32 (clone 93, eBioscience) to block Fc receptors, then stained with antibodies to surface molecules and washed two times in FACS buffer (PBS with 0.5% w/v BSA). Flow cytometry antibody clones, fluorochromes, concentrations, vendors and catalogue numbers for mouse cells (Supplementary Table 1) and human cells (Supplementary Table 2) can be found in the Supplementary Materials online. For preparation of single-cell suspensions from islet grafts, grafts were exposed by partially peeling the kidney capsule from the kidney surface, removed, digested with collagenase P (1.5 mg ml^−1^) at 37 °C in a water bath for 5 min, and then made into a single-cell suspension by passing through 70-μm nylon mesh screens (Fisher Scientific). For detection of LT by flow cytometry, cells were incubated with MOPC21 or LTβRIg at 2 μg ml^−1^ for 60 min at 37 °C in HBSS (Lonza), 2% FBS (Gemini), 1 mM sodium pyruvate (Lonza), 2 mM L-glutamine (Lonza), 100 IU ml^−1^ penicillin (Lonza), 100 μg ml^−1^ streptomycin (Lonza), 1 × non-essential amino acids (Lonza) at no more than 5 × 10^6^ cells per ml. Cells were washed twice in the HBSS solution, then stained for 45 min with surface antibodies including Rat anti-Mouse IgG1κ at 200 ng ml^−1^ for 45 min at 4 °C. Cells were then washed twice in HBSS buffer, and were fixed with 4% paraformaldehyde at room temperature in the dark for 10 min, washed, run on an LSR Fortessa flow cytometer (BD Biosciences), and results analysed with FlowJo 8.7 (Treestar).

When appropriate, intracellular staining for Foxp3 followed the extracellular step using eBiosciences Foxp3 intracellular staining buffer. ef450 fixable viability dye (eBiosciences) was used according to the manufacturer's protocol. Cells were then fixed, processed and analysed as above. For SVEC4-10 flow cytometry 2 × 10^4^ SVEC4-10 cells were cultured in a 24-well plate overnight, medium was removed and replaced with 0.5 ml medium containing agonistic anti-LTβR (AFH6 1 μg ml^−1^, Biogen Idec) or control mAb (Ha4/8 1 μg ml^−1^, BD, 553961) for 24 h. At that time cells were collected for flow cytometry.

### Real-time PCR

Primer sequences were: *Foxp3* forward 5′-CCCAGGAAAGACAGCAACCTT-3′ and reverse 5′-TTCTCACAACCAGGCCACTTG-3′; *Ltbr* forward 5′-TTGCTGGGACCAGGACAAGGAATA-3′ and reverse 5′-GGCTGCATACCGCAAAGACAAACT-3′; *Ccr7* forward 5′-CACGCTGAGATGCTCACTGG-3′ and reverse 5′-CCATCTGGGCCACTTGGA-3′; *S1pr1* forward 5′-GTGTAGACCCAGAGTCCTGCG-3′ and reverse 5′-AGCTTTTCCTTGGCTGGAGAG-3′; *Il10* forward 5′-AGTGCCTGGTTGCTGCTTTACC-3′ and reverse 5′-AGCCGTAGGGTCTTTAGATTTTCAC-3′; *Tgfb1* forward 5′-CTGCTGAGGCTCAAGTTAAAAGTG-3′ and reverse 5′-CAGCCGGTTGCTGAGGTAG-3′; *Prf1* forward 5′-GAGAAGACCTATCAGGACCA-3′ and reverse 5′-AGCCTGTGGTAAGCATG-3′; *Gzmb* forward 5′-CCTCCTGCTACTGCTGAC-3′ and reverse 5′-GTCAGCACAAAGTCCTCTC-3′.

*Ifng* forward 5′-CTTTGGACCCTCTGACTTGAG-3′ and reverse 5′-TCAATGACTGTGCCGTGG-3′; *Bcl2* forward 5′-CTGGCATCTTCTCCTTCCAG-3′ and reverse GACGGTAGCGACGAGAGAAG-3′; *Bcl2l11(Bim)* 5′-GAGATACGGATTGCACAGGA-3′ and revserse 5′-TCAGCCTCGCGGTAATCATT-3′; *Ppia (Cyclophilin A)* forward 5′-AGGGTGGTGACTTTACACGC-3′ and reverse 5′-ATCCAGCCATTCAGTCTTGG-3′; *Cd39* forward 5′-CGAGAAGGAGAATGACACAGG-3′ and reverse 5′-ATGTTGGTATCAGTTCGGTGG-3′; *Cd73* forward 5′-GCTTCAGGGAATGCAACATG-3′ and reverse 5′-TGCCACCTCCGTTTACAATG-3′; *Ctla4* forward 5′-CATGCCCGGATTCTGACTTC-3′ and reverse 5′-GGCATTTTCACATAGACCCCTG-3′; *Tnfrsf14/Hvem* forward 5′-TGAAGCAGGTCTGCAGTGAGCATA-3′ and reverse 5′-GCCATTTGCATGGGCGGTATATGT-3′; *Lta* forward 5′-TGAAACCTGCTGCTCACCTTGTTG-3′ and reverse 5′- AAGAGAAGCCATGTCGGAGAAAGG-3′; *Ltb* forward 5′-TCCAATGCTTCCAGGAATCTAGCC-3′ and reverse 5′-GACAGGATGGTCATCCATGCAGAT-3′; and *Tnfsf14/LIGHT* forward 5′- AAAGGCTCCTGGGAGAAGCTGATA-3′ and reverse 5′-ATACCTATCAAGCTGGCGTTGGCT-3′. Total cellular RNA was extracted from the indicated T cell and endothelial cell populations using Trizol reagent (Invitrogen), digested with RNase-free DNase I to remove any contaminating genomic material (Invitrogen), and reverse-transcribed into cDNA using Omniscript RT Kit (Qiagen) and random primers (Invitrogen) according to the manufacturer's protocol. mRNA expression levels were quantified by real-time PCR using Quantitect SYBR Green PCR kit (Qiagen) with an ABI Prism 7900HT (Applied Biosystems). PCR consisted of a 15-min 95 °C denaturation step followed by 45 cycles of 15 s at 94  °C, 20 s at 56 °C and 20 s at 72 °C. Values for specific gene mRNA expression as a % of cyclophilin A were calculated as: 2^(Ct of Cyclophilin A−Ct specific gene)^ × 100.

### Murine islet isolation and transplantation

The following procedure has been adapted from a published protocol^53^.

*Reagents*. EuroCollins solution was prepared by adding 20 ml electrolyte additive solution (Catalogue No: 99-409-CI, Cellgro) to 1 l Glucose Solution (Catalogue No: 99-408-CM, Cellgro). Ficoll 400-DL, (Catalogue No. F9378, Sigma) solutions were prepared at concentrations of 25, 23, 21 and 11% (w/v) in EuroCollins solution. Lower concentrations of Ficoll solution were diluted from 25% stock. Collagenase P (Catalogue No. 11213873001, Roche) solution was prepared at 1.5 mg ml^−1^ in HBSS with Ca2+ and Mg2+ (Catalogue No. 12-020-CV, Cellgro), with 2 mg ml^−1^ BSA (Catalogue No. A7906, Sigma). RPMI 1640 (Catalogue No. 12-702F, Lonza) with 10% FBS (Benchmark, Gemini) was also prepared.

*Injection*. Just before dissection, BALB/c mice were killed by CO_2_ inhalation followed by cervical dislocation. The entire mouse was with 70% ethanol and a V-shaped incision was made starting at the genital area and ending near the ribcage. Mice were oriented with the tail facing away from the experimenter and intestines were moved aside to expose the pancreas and common bile duct. A haemostat clamp was placed on the duodenum just posterior of the ampulla, where the bile duct drains, leaving a small space for collagenase P solution to enter the duodenum.

A 30-g needle on a 3-ml syringe containing 3 ml of cold collagenase P solution was prepared, and the needle inserted into the common bile duct at the joint site of the hepatic duct and the cystic duct. The pancreas was then inflated by slowly but steadily injecting the solution. Next, the pancreas was removed and placed in a 50-ml tube containing 2 ml of collagenase P solution on ice. With a maximum of 2.5–3 pancreata per 50-ml tube. This step was done as quickly as possible.

*Digestion*. Note: Islets stick to plastic, so all plastic surfaces that will contact islets must be pre-coated with RPMI 1640/10% FBS!

The 50-ml tubes with pancreata were placed in 37 °C water bath for 13–15 min. After incubation, tubes were shaken by hand to disrupt the pancreas until the suspension was homogeneous. Next, cold RPMI 1640/10% FBS was added to the tube to bring the total volume to 50 ml. Tubes were centrifuged at 70*g* for 1 min and the supernatant was aspirated leaving ∼5 ml to avoid disturbing the pellet. A Cellector Tissue Sieve (Bellco Glass) was placed in a funnel over a 50-ml conical tube and the sieve was wetted with 20 ml of RPMI 1640/10% FBS. The pellet was then re-suspended in 20 ml RPMI 1640/10% FBS and poured through sieve into a 50-ml tube and the screen was washed with 10 ml RPMI 1640/10% FBS. Tubes were then centrifuged at 90*g* for 1 min. Supernatant was aspirated the pellet was re-suspended by tapping with the hand, then RPMI 1640/10% FBS was added up to 50 ml. Pellets were washed in this manner two more times. Finally, the supernatant was aspirated as completely as possible. (as remaining buffer might cause the change of the density of the Ficoll.)

*Ficoll gradient enrichment*. Perform this process quickly. Long-term Ficoll exposure is toxic to islets.

The 50-ml tubes were tapped on the bottom by hand to ensure pellet was completely dissociated before adding Ficoll as unbroken tissue is difficult to re-suspend in dense, viscous Ficoll. Pellet was then re-suspended in 10 ml of Ficoll 25% by finger tapping. Remaining Ficoll densities were then layered on top using a 5-ml pipette in this order: 23, 21 and 11%. Ficoll gradient preparations were centrifuged at 600*g* at 4 °C, with no brake for 15 min. If centrifuge allows control over acceleration rate, set in the middle of the range. Islets were picked from the interface of the second and third layers using a bulb pipette and transferred to a 50-ml tube containing ∼25 ml of cold RPMI 1640/10% FBS. Islet pellets were washed twice, centrifuging at 270*g* for 4 min and 188*g* for 3 min using the brake. Islet pellets were then re-suspended in 20 ml RPMI 1640/10% FBS.

*Islet picking*. Islets were transferred islets to a 100-mm Petri dish and were hand-picked using an inverted light microscope and a 200 μl pipette, and transferred into a 1.7-ml Eppendorf tube and kept on ice until ready for transplantation.

*Transplantation*. A total of 400 freshly isolated islets were hand-picked and transplanted beneath the renal capsule of C57BL/6 recipients made diabetic (blood glucose >300 mg dl^−1^) by intraperitoneal injection of 180 mg kg^−1^ streptozotocin (Sigma-Aldrich) at least 1 week before transplant. nTregs were purified with anti-CD25 PE magnetic bead positive selection (Miltenyi) from C57BL/6 donor LN and spleens or *Ltα*^−/−^ donor spleens. Cells were then labelled with efluor 670+ at 5 μM (eBioscience) or CFSE at 5 μM (Invitrogen) according to the manufacturer instructions, and 1 × 10^6^ magnetic bead-purified CD25+ nTreg were injected either with islets under the kidney capsule (local transfer) or i.v. Blood glucose <150 mg dl^−1^ after transplantation was considered engraftment, and >300 mg dl^−1^ was considered diabetic or graft rejection as appropriate. Blood glucose was monitored via tail nicking with a handheld meter (Therasense).

### Footpad and whole-mount ear migration assays

CD4+ T cells were enriched by magnetic bead negative selection (Stemcell Technologies). CD25^hi^ Tregs were further purified using anti-CD25-PE, anti-PE microbeads and magnetic separation (Stemcell Technologies), then labelled with CFSE (Life Technologies). T cells were treated with LTβRIg or MOPC21 at 50 μg ml^−1^ as indicated for 30 min at 37 °C and then washed 2 × with PBS. In some experiments 2.5 μg of NA/LE grade anti-VCAM-1 (429, BD, 553329) or NA/LE Rat IgG2a isotype control (clone R35-95 BD, 554687) were co-injected with T cells. In other experiments T cells were pretreated with NA/LE grade anti-VLA-4 (clone R1-2, BD, 553153), NA/LE grade Rat IgG2b (clone R35-38, BD,555845) at 10 μg ml^−1^ for 30 min at 4 °C and washed 3 × with PBS before injection. Mice were anaesthetized and 1 × 10^6^ CD25+ Tregs or CD4+ non-Tregs were injected into the footpads or ear pinnae in 20 μl PBS. For footpad assays, draining popliteal LN were collected 12 h post injection and processed for flow cytometry. For ear pinnae assays, whole-mount ears were collected 4 h post injection, fixed for 10 min at room temperature with 4% paraformaldehyde, then stained with anti-LYVE-1 at 5 μg ml^−1^ (Fitzgerald, 70R-LR003), anti-CCL21 at 5 μg ml^−1^ (R&D Systems, AF457) in PBS 0.5% BSA 0.3% Triton X-100 overnight at 4 °C, washed with PBS 0.5% BSA 0.3% Triton X-100, stained with Donkey anti-Rabbit Cy3 at 5 μg ml^−1^ (Jackson ImmunoResearch, 711-165-152) and Donkey anti-Goat Alexa Fluor 488 at 5 μg ml^−1^ (Jackson ImmunoResearch, 705-546-147) for 2 h at room temperature, washed 2 × in PBS 0.5% BSA 0.3% Triton X-100, fixed once more, transferred to glass slides and mounted with Prolong Gold (Life Technologies).

### LN egress assay

We adapted a procedure previously reported to determine T-cell egress rates from LNs^54^. A mixture of 5 × 10^6^ WT C57BL/6 eFluor 670+ CD4 T cells and 5 × 10^6^ CFSE+ *Ltα*^−/−^ CD4 T cells was injected i.v. into naive C57BL/6 mice. After 18 h 100 μg anti-CD62L (MEL-14, BioXCell) or control Rat IgG2a (2A3, BioXCell) was injected i.v. After 18 h of antibody injection LN and spleen were collected and analysed by flow cytometry.

### Statistical analysis

No statistical tests were used to predetermine the size of experiments. Sample and experiment sizes were determined empirically for sufficient statistical power. No samples were excluded specifically from analysis, and no randomization or blinding protocol was used. Survival curves were analysed according to the Kaplan–Meier estimator, and the difference between two groups was determined by the log-rank (Mantel–Cox) test. Log-rank, two-tailed Student's *t*-tests, Mann–Whitney non-parametric tests, one-way analysis of variance with Tukey, Bonferroni post tests or Kruskal–Wallis test with Dunn's post tests were performed using Graphpad software (Prism V5.0). Mann–Whitney and Kruskal–Wallis tests were used when data were non-Gaussian due to presence of outliers or data clustered at a limit of detection. For multiple comparisons, Tukey post tests were used when all possible comparisons were logically meaningful, and Bonferroni or Dunn's post tests were used when only certain pairs of comparisons were experimentally or logically possible. A *P* value of <0.05 was considered significant for one-way analysis of variance and *t*-tests, and for multiple comparisons significance level was adjusted for the number of comparisons being made by the software. Statistical analysis was performed on groups with similar variance. Error bars in figures and value ranges given in the text are mean±s.e.m. unless otherwise noted.

### Image adjustments

Images were adjusted for contrast and brightness over the entire field to improve visibility.

### Study approval

All animal experiments were performed in accordance with Institutional Animal Care and Use Committee approved protocols.

### Data availability

The data that support the findings of this study are available from the corresponding author on request.

## Additional information

**How to cite this article:** Brinkman, C. C. *et al*. Treg engage lymphotoxin beta receptor for afferent lymphatic transendothelial migration. *Nat. Commun.* 7:12021 doi: 10.1038/ncomms12021 (2016).

## Supplementary Material

Supplementary Information Supplementary Figures 1-6 and Supplementary Tables 1 and 2

Supplementary Movie 1 MOPC21 treated wild type nTreg movement in ears.

Supplementary Movie 2 LTβRIg treated wild type nTreg movement in ears.

Supplementary Movie 3 MOPC21 treated Lta -/- nTreg movement in ears

Supplementary Movie 4 LTβRIg treated Lta-/- nTreg movement in ears

Supplementary Movie 5 RatIgG2a treated wild type nTreg movement in ears.

Supplementary Movie 6 Anti-VCAM-1 treated wild type nTreg movement in ears.
