# Supplementary material for: Treg engage lymphotoxin beta receptor for afferent lymphatic transendothelial migration
Source: Nat Commun. 2016 Jun 21;7:12021. doi: 10.1038/ncomms12021 (PMC4919545; doi:10.1038/ncomms12021)
Supplement: Supplementary Information — Supplementary Figures 1-6 and Supplementary Tables 1 and 2 [file ncomms12021-s1.pdf]

Supplementary Figure 1

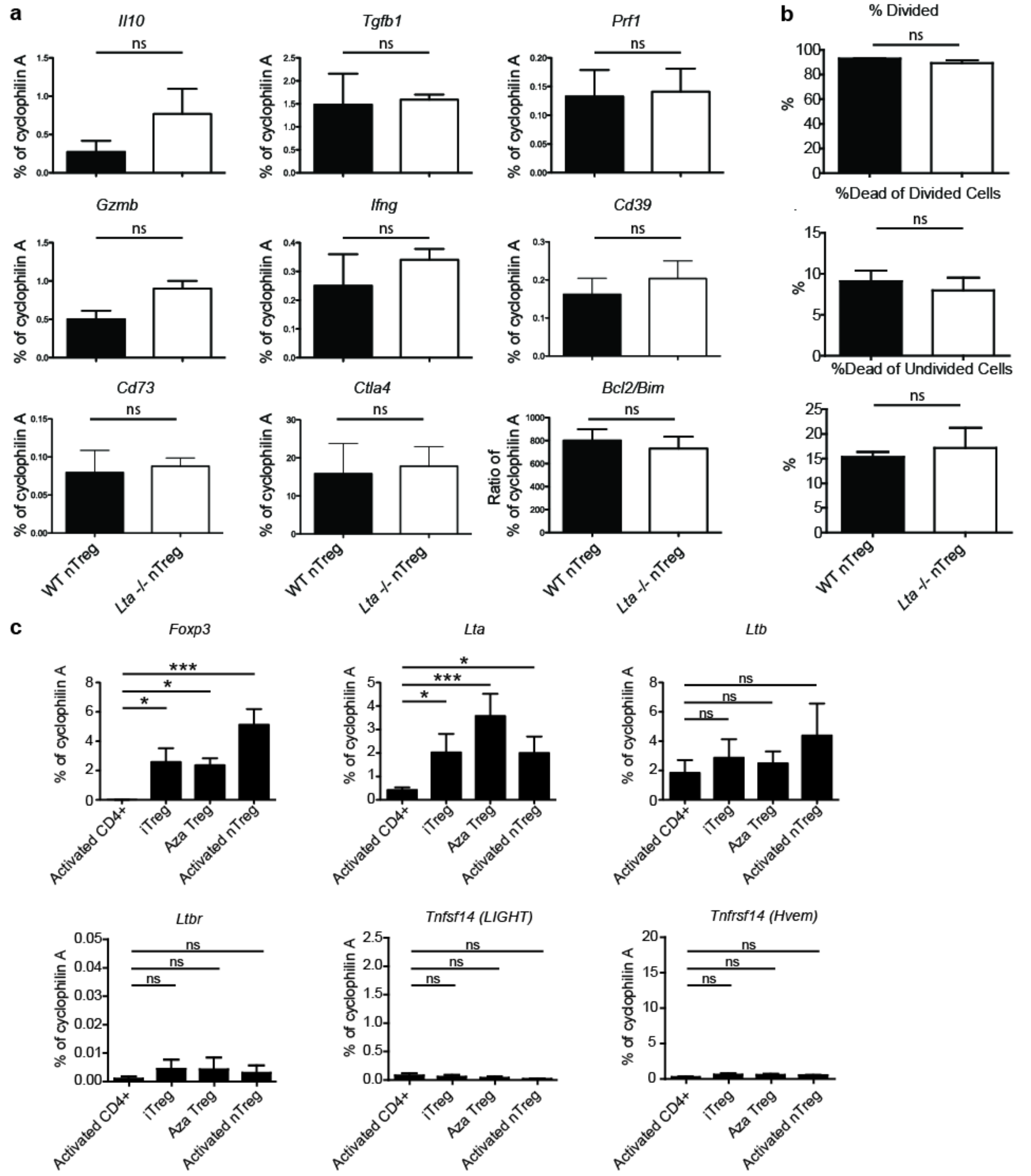

Supplementary Figure 1. Wild type and *Lta -/-* Treg do not differ in expression of effector and survival related genes. (a) qRT-PCR data from nTreg. Y-axes show indicated gene expression as % of cyclophilin A. Values from triplicate wells per experiment, 1 mouse per experiment, 3 experiments pooled. (b) Summary of proliferation and survival from CD4+CD25hi wild type or *Lta* $^{-/-}$ nTreg stimulated with anti-CD3, anti-CD28, and IL-2 for 5 days. Treg gated on CD4+CD25+Foxp3+. Proliferation assessed by CFSE dilution compared to unstimulated controls and viability assessed using ef450 fixable viability dye. Error bars are mean +-SEM. Results from triplicate wells from one experiment representative of two experiments. Statistics from two-tailed unpaired Student's t tests. (c) qRT-PCR from in vitro generated T cell subsets. Y-axes indicate expression as % of cyclophilin A. Data pooled from 6-7 experiments for *Foxp3*, *Lta* and *Ltb*, 4-5 for remaining targets. * $p<0.05$, ** $p<0.01$, *** $p<0.001$ P values from Dunn's multiple comparison test. Error bars are mean +-s.e.m.

Supplementary Figure 2

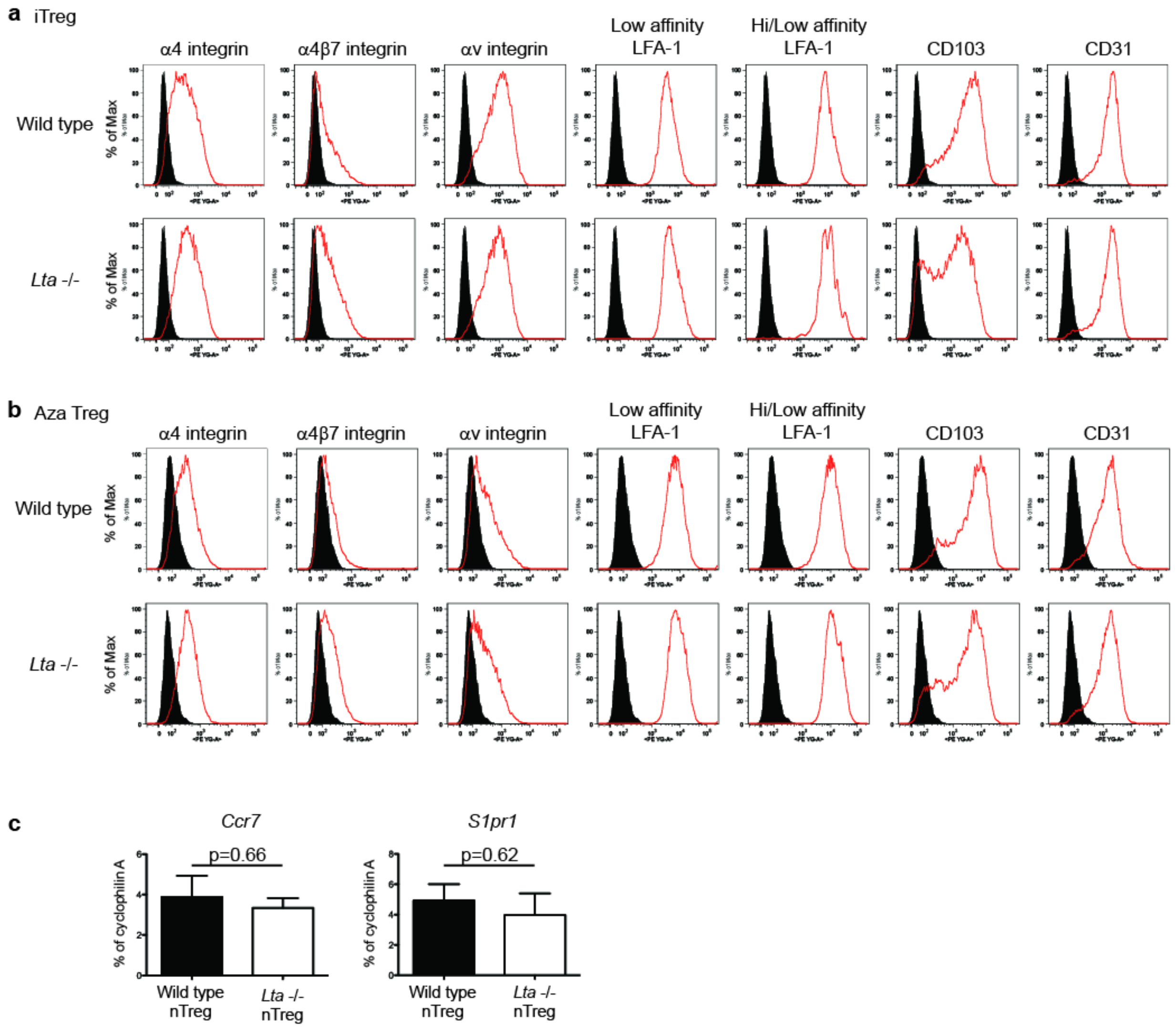

Supplementary Figure 2. **Wild type and *Lta -/-* Treg do not differ in expression of multiple migration molecules.** (a, b) Indicated T cell subsets generated and analyzed by flow cytometry. Treg gated on CD4+CD25+Foxp3+ and stained for indicated surface molecules. Isotype control, black histograms; indicated antibody, red histograms. Results from 1 experiment representative of 2. (c) qRT-PCR data from nTreg. Y-axes show indicated gene expression as % of cyclophilin A. Results from mean of triplicate reactions from 1 mouse per experiment, average of 3 experiments. P values from two tailed unparied Student's t tests. Error bars are mean +-s.e.m.

Supplementary Figure 3

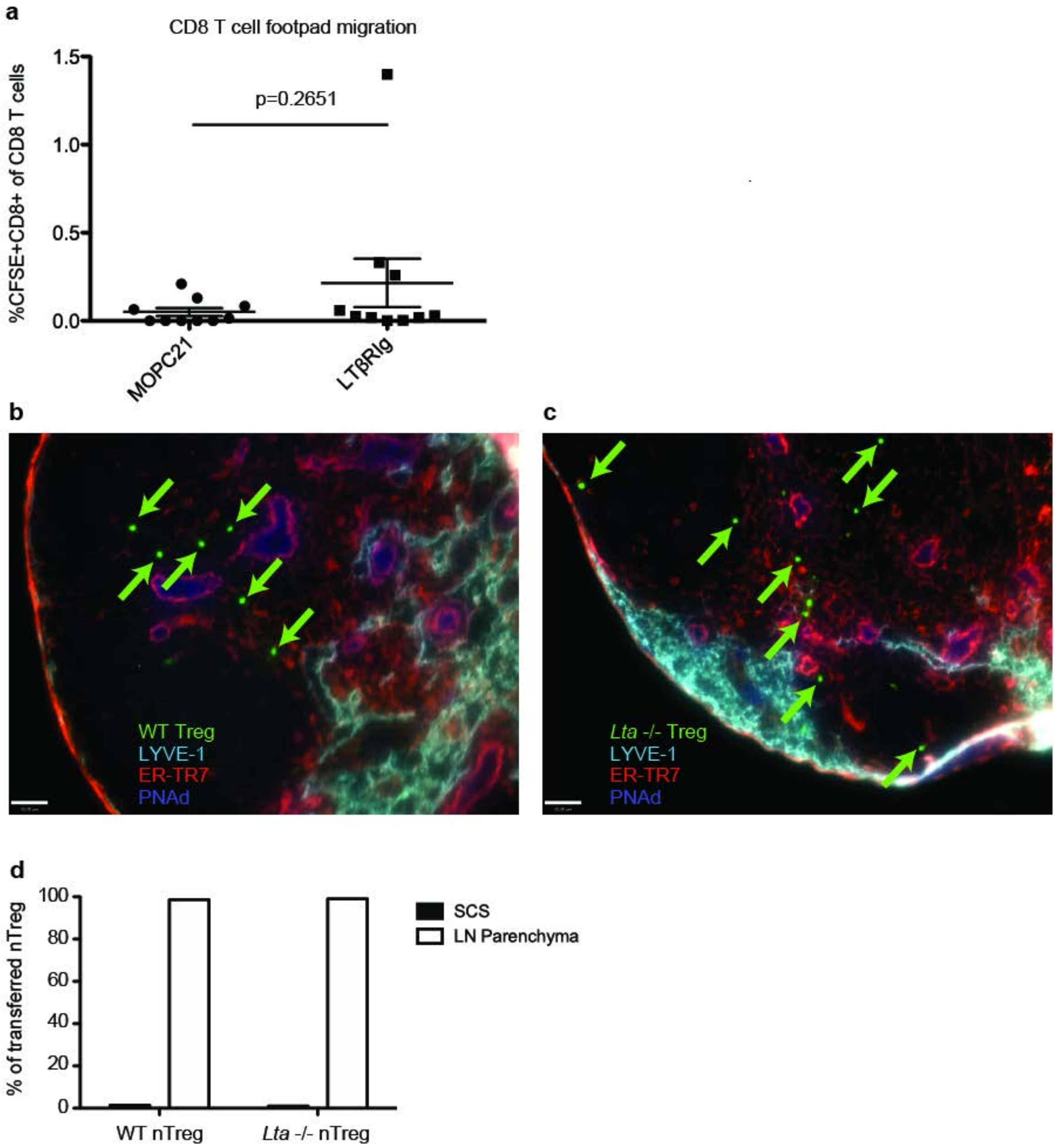

Supplementary Figure 3. WT and *Lta -/-* Treg do not differ in their localization within LN after migration from the footpad. (a) CD8 T cells treated with control MOPC21 or LTβRIg, washed, and injected into footpads. Organs procured and analyzed by flow cytometry. Results shown as % transferred CD8 T cells (a) of total LN CD8 T cells. (b, c) Representative fluorescent microscope images showing WT (c) or *Lta* $^{-/-}$ (d) Treg in popliteal LN. CFSE+ Treg in green, LYVE-1 in light blue, ER-TR7 in red, and PNAd in dark blue. 20x objective. Scale bar = 42µm. (d) Summary data from 15 images from 9 sections derived from 3 LN from 1 experiment showing WT and *Lta* $^{-/-}$ nTreg localization within popliteal LN, representative of two experiments. P values from Mann-Whitney non-parametric tests from 10 mice per condition from 2 experiments in (a) Error bars are mean +-s.e.m.

Supplementary Figure 4

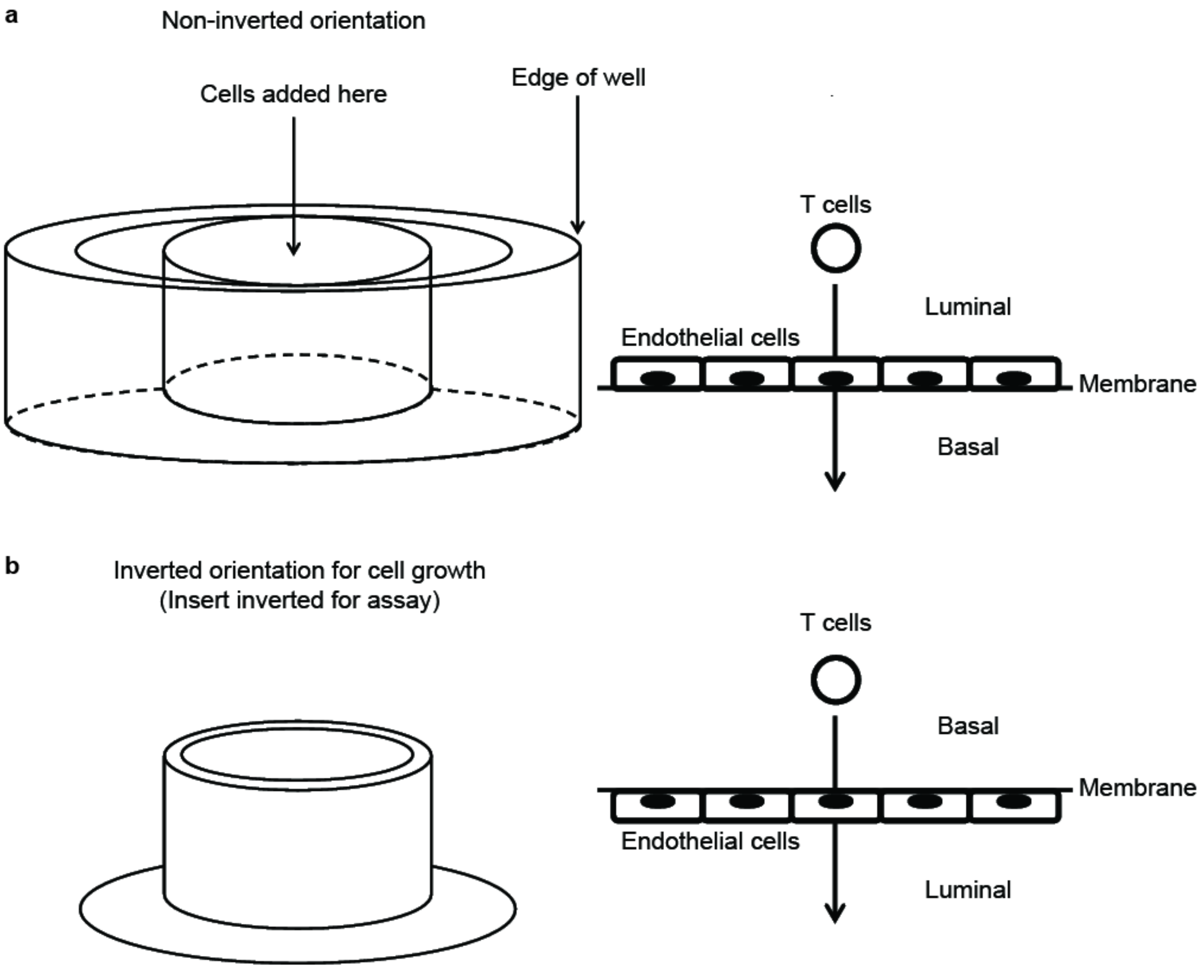

Supplementary Figure 4. Culture of endothelial cells. (a) Orientation of transwell inserts during initial culture of endothelial cells in inverted position (iSVEC4-10 or iMS-1). (b) Orientation of transwell inserts during culture of non-inverted (SVEC4-10 or MS-1). (c) Orientation of inserts during transmigration or basal structure growth.

Supplementary Figure 5

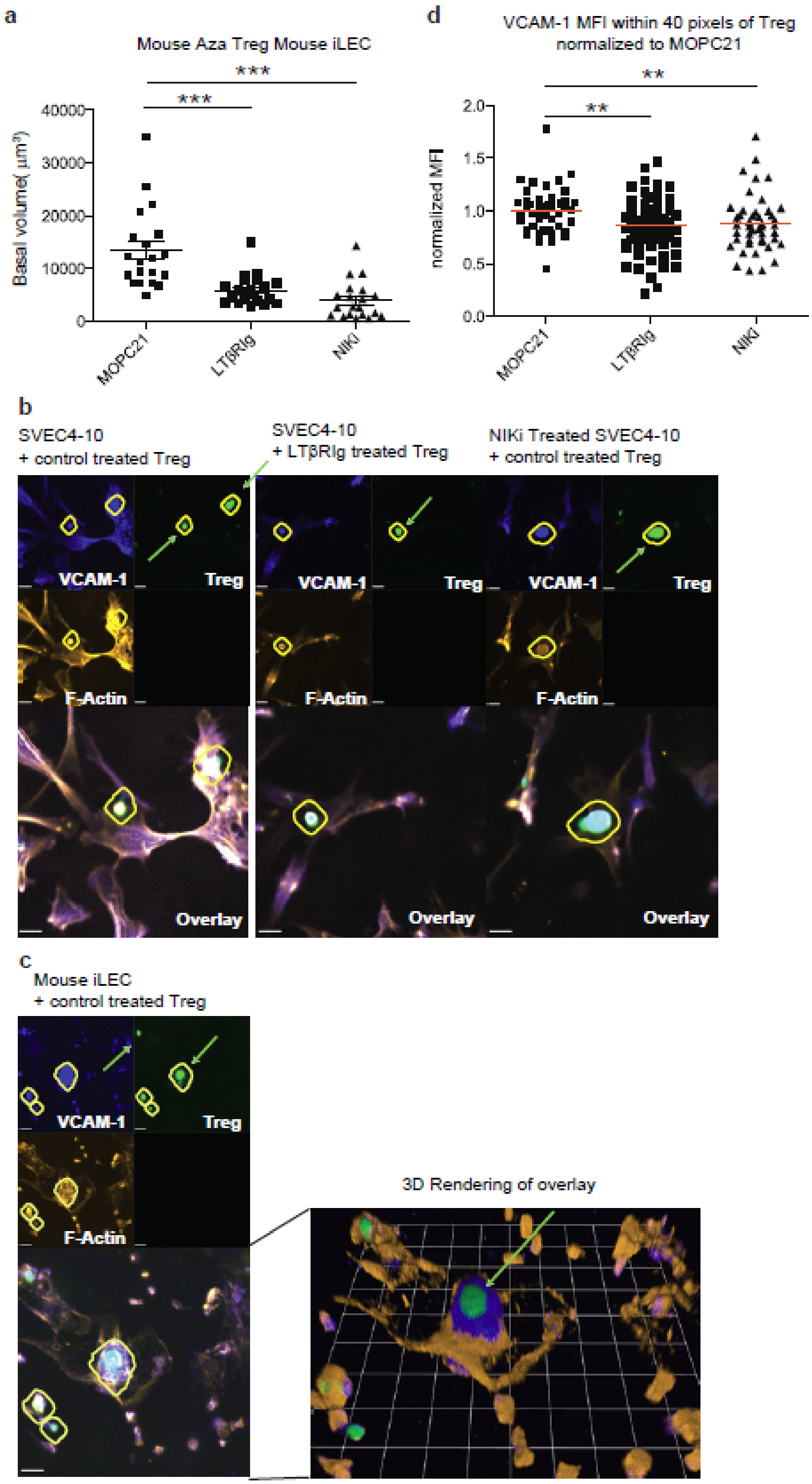

Supplementary Figure 5. Characterization of basal lamellipodia like protrusions on iSVEC4-10 and mouse iLEC layers. (a) Summary data showing volume of basal phalloidin+ staining area volume for primary mouse iLEC layers incubated with WT mouse Aza Treg in presence of indicated treatments. Results from 20-21 images from 4 inserts from 2 independent experiments. (b-c) Representative confocal images from transwell migration experiments of mouse Aza Treg across iSVEC4-10 (b) or primary mouse iLEC (c). VCAM-1 in blue; CFSE+ Aza Treg in green; phalloidin+f-actin in orange. 40x objective. Green arrows point to Treg in contact with basal lamellipodia-like protrusions. Yellow regions mark 40 pixel regions around Treg used for MFI analysis. Scale bar 10µm. In (c) channel overlay panel is also shown using a 3d rendering. (d) Summary data from 45-67 images from 5-6 inserts from 3 independent experiments of WT Aza Treg migrating through iSVEC4-10 to CCL19, MFI normalized to MOPC21 control in each experiment. VCAM-1 MFI within 40 pixels of Treg. ** $p<0.01$, *** $p<0.001$ by Dunn's multiple comparison post-test of Kruskal-Wallis Gaussian approximation. Error bars are mean +-s.e.m.

Supplementary Figure 6

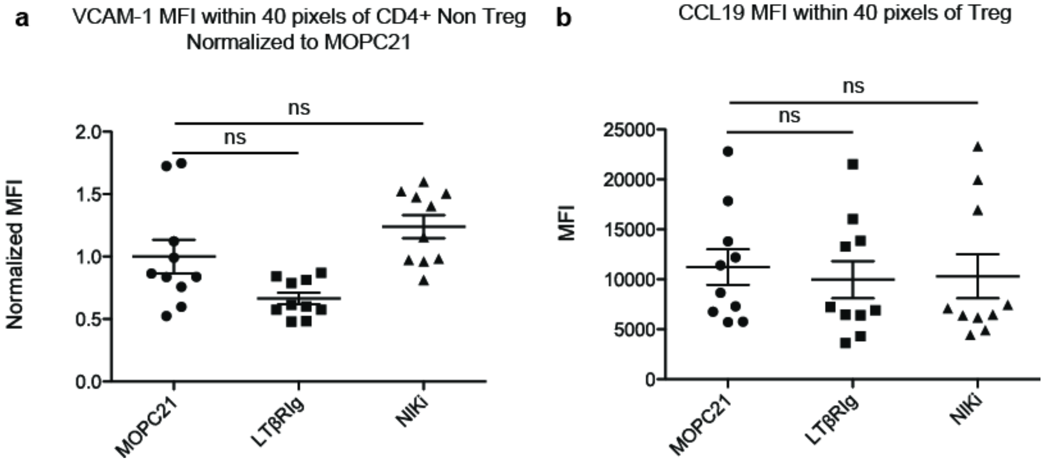

Supplementary Figure 6. Characterization of basal lamellipodia like protrusions on iSVEC4-10 layers. (a) Summary data showing VCAM-1 MFI within 40 pixels of CD4+ non Treg under indicated conditions. Results from 10 images from 2 inserts per condition from 1 experiment. (b) Summary data showing CCL19 MFI within 40 pixels of Treg under indicated conditions. Results from 20 images from 1 insert each from 2 independent experiments . * $p<0.05$, ** $p<0.01$, *** $p<0.001$ by Dunn's multiple comparison test. Error bars are mean +- s.e.m.

Supplementary Table 1. Murine Flow Cytometry Antibodies

Specificity	Clone	Fluorochrome	Catalogue #	Dilution or Concentration	Vendor
CD16/32 ("FC block")	93	Unconjugated	14-0161	1:250	eBioscience
αV integrin (CD51)	RMV-7	PE	12-0512	1:250	eBioscience
α4 integrin (CD49d)	R1-2	PE	12-0492	1:250	eBioscience
α4β7 integrin	LPAM-1	PE	12-5887	1:250	eBioscience
CD103	2E7	PE	12-1031	1:250	eBioscience
CD31	390	PE, APC	12-0311	1:250	eBioscience
CD11a low affinity form	2D7	PE	553121	1:250	BD
CD11a high/low affinity form	M17/4	PE	12-0111	1:250	eBioscience
CD4	GK1.5	APC-eFluor 780	47-0041	1:500	eBioscience
CD8	53-6.7	APC	17-0081	1:500	eBioscience
CD44	IM7	PE-Cy7	25-0441	1:500	eBioscience
CD25	PC61.5	PE,<br>PE-Cy7	12-0251,<br>25-0251	1:250	eBioscience
FoxP3	FJK-16s	FITC,<br>PE,<br>PE-Cy7	11-5773,<br>12-5773,<br>25-5773	1:100	eBioscience
VCAM-1	429	Alexa Fluor 647	51-1061	1:200	eBioscience
ICAM-1	YN1	FITC	11-0541	1:200	eBioscience
E-selectin (CD62E)	10E9.6	PE	553751	1:200	BD
P-selectin (CD62P)	RB40.34	FITC	561923	1:200	BD
Unknown	MOPC21	unconjugated	BE008	2µg/ml	BioXcell
LTα1β2	LTβRIg	unconjugated	N/A	2µg/ml	Biogen
Mouse IgG1κ	RMG1-1	Brilliant Violet 421	406616	200ng/ml	Biolegend
LTβR	eBio3C8	PE	12-5671	2µg/ml	eBioscience

Supplementary Table 2. Human Flow Cytometry Antibodies

Specificity	Clone	Fluorochrome	Catalogue #	Dilution	Vendor
CD4	RPA-T4	APC-ef780	47-0049-42	1:20	eBioscience
CD127	eBioRDR5	PerCP-Cy5.5	45-1278-42	1:20	eBioscience
CD45RA	HI100	PE-Cy7	25-0458-42	1:20	eBioscience
FoxP3	259D	Pacific Blue	320216	1:20	Biolegend
Helios	22F6	FITC	137214	1:100	Biolegend
